# Multi‐material Volumetric Bioprinting and Plug‐and‐play Suspension Bath Biofabrication via Bioresin Molecular Weight Tuning and via Multiwavelength Alignment Optics

**DOI:** 10.1002/adma.202409355

**Published:** 2025-02-26

**Authors:** Davide Ribezzi, Jan‐Philip Zegwaart, Thomas Van Gansbeke, Aitor Tejo‐Otero, Sammy Florczak, Joska Aerts, Paul Delrot, Andreas Hierholzer, Martin Fussenegger, Jos Malda, Jos Olijve, Riccardo Levato

**Affiliations:** ^1^ Department of Orthopaedics University Medical Center Utrecht Utrecht University Utrecht 3584 CX The Netherlands; ^2^ Rousselot Port Arthurlaan 173 Gent 9000 Belgium; ^3^ Department of Clinical Sciences Faculty of Veterinary Medicine Utrecht University Utrecht 3584 CT The Netherlands; ^4^ BIOMAT Research Group University of the Basque Country (UPV/EHU) Escuela de Ingeniería de Gipuzkoa Plaza de Europa 1 Donostia‐San Sebastián 20018 Spain; ^5^ Readily3D SA EPFL Innovation Park, Building A Lausanne CH‐1015 Switzerland; ^6^ Department of Biosystems Science and Engineering ETH Zurich Mattenstrasse 26 Basel CH‐4058 Switzerland; ^7^ Faculty of Science University of Basel Mattenstrasse 26 Basel CH‐4058 Switzerland

**Keywords:** biofabrication, embedded printing, hydrogels, pancreas tissue engineering, volumetric additive manufacturing

## Abstract

Volumetric Bioprinting (VBP), enables to rapidly build complex, cell‐laden hydrogel constructs for tissue engineering and regenerative medicine. Light‐based tomographic manufacturing enables spatial‐selective polymerization of a bioresin, resulting in higher throughput and resolution than what is achieved using traditional techniques. However, methods for multi‐material printing are needed for broad VBP adoption and applicability. Although converging VBP with extrusion bioprinting in support baths offers a novel, promising solution, further knowledge on the engineering of hydrogels as light‐responsive, volumetrically printable baths is needed. Therefore, this study investigates the tuning of gelatin macromers, in particular leveraging the effect of molecular weight and degree of modification, to overcome these challenges, creating a library of materials for VBP and Embedded extrusion Volumetric Printing (EmVP). Bioresins with tunable printability and mechanical properties are produced, and a novel subset of gelatins and GelMA exhibiting stable shear‐yielding behavior offers a new, single‐component, ready‐to‐use suspension medium for in‐bath printing, which is stable over multiple hours without needing temperature control. As a proof‐of‐concept biological application, bioprinted gels are tested with insulin‐producing pancreatic cell lines for 21 days of culture. Leveraging a multi‐color printer, complex multi‐material and multi‐cellular geometries are produced, enhancing the accessibility of volumetric printing for advanced tissue models.

## Graphical abstract

Multimaterial volumetric and embedded extrusion bioprinting of complex cell‐laden architectures is enabled by unravelling the interplay between molecular weight and degree of functionalization in gelatin‐based bioresins. Tomographic light‐responsive support baths for suspended printing can be easily generated from single component materials. Molecular weight tuning produces a library of printable hydrogels matching the viscoleasticity properties of native tissues, influencing cell self‐organization.

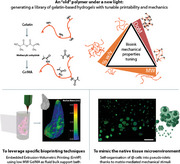

## 1 Introduction

Recent advances in 3D printing technologies led to the emergence of volumetric 3D printing, also termed volumetric additive manufacturing, a new family of techniques capable of producing objects of virtually any shape and size in a matter of seconds.^[^ ^1^, ^2^, ^3^ ^]^ With a specific focus on the field of biofabrication, the development of Volumetric Bioprinting (VBP) enabled to sculpting of hydrogels and living cells into centimeter‐scale, clinically‐relevant sized constructs at unprecedented velocity.^[^ ^3^ ^]^ This opens up new avenues for large‐scale tissue engineering and regenerative medicine, as well as for the high‐throughput production of advanced in vitro tissue models for biomedical and pharmaceutical research.^[^ ^3^, ^4^ ^]^ While several volumetric printing modalities have been introduced,^[^ ^5^, ^6^, ^7^, ^8^ ^]^ the fastest approach to date to build convoluted porous geometries typical of living tissues, relies on the principles of tomographic manufacturing. In this respect, VBP is performed by projecting visible light from a spatial light modulator (i.e., a digital micromirror device, DMD) onto a rotating vial containing a photoresponsive, cell‐laden hydrogel, also termed bioresin. At each angle of rotation, the DMD delivers a different 2D projection, calculated following a tomographic reconstruction algorithm. Altogether, the light projections induce a cumulative light dose exceeding the threshold of cross–linking of the bioresin only in the voxels that correspond to the object to be printed, which solidifies in a layer‐less fashion. This mode of printing results in much faster (<15 s) fabrication rates compared to layer‐by‐layer extrusion and digital light processing (DLP) or stereolithographic (SLA) printing, while achieving resolutions in the range of 40 µm, and allowing for a safe process of fragile cells and organoids in absence of mechanical stresses.^[^ ^4^ ^]^

Nevertheless, for VBP to be broadly adopted, key challenges remain to be overcome. First, tomographic printing introduces new requirements in terms of material properties (optical, rheological) for the bioresins. In particular, accurate tuning of the photocross–linking kinetics is paramount to producing accurate prints and avoiding excessive light dose accumulation in non‐desired regions of the bioresin vial. Moreover, methods for facile multi‐material printing and for the production of complex tissues comprising multiple cell populations are needed. We recently introduced the concept of Embedded extrusion Volumetric Printing (EmVP), a technique converging suspended bath bioprinting, as a means to position multiple cell types in a bioresin vat, and VBP to sculpt the now isotropic bioresin into any desired geometry. This method relied on the use of photoannelable microparticles (or microgels) as bioresin. This granular composition, prior to photocross–linking, acts as a Bingham‐like material suitable as a bath for suspended printing.^[^ ^9^ ^]^ While versatile, this approach limits the printing resolution to the size of the microgels. Other groups later explored the use of gelatin as a sacrificial, and thermoreversible viscosity enhancer blended with other resins of interest.^[^ ^10^ ^]^ However, this approach is challenged by limited printing resolution and a short time window of printability, since gelatin typically displays an ideal temperature‐dependent, shear thinning behavior, restricted in a narrow (few minutes only) range during its thermal gelation curve.^[^ ^10^ ^]^

Herein, we propose that engineering the gelatin macromers will allow to overcome these challenges, and, on top of establishing a library of GelMA resins with a wide range of mechanical properties, we identified a fit‐for‐purpose formulation for suspended bath printing and EmVP. Notably, gelatin, and particularly its photocross–linkable derivative gelatin methacryloyl (GelMA), has become a golden standard material in the field of bioprinting. This is due to their availability in large amounts, biocompatibility, content of integrin‐binding domains (i.e., RGD sequences), and intrinsic capacity to sustain cell adhesion and proliferation.^[^ ^11^, ^12^ ^]^ Despite the relevant body of literature concerning these materials, key polymer phyisco‐chemical properties, such as molecular weight,^[^ ^13^, ^14^, ^15^ ^]^ are mostly overlooked. If properly tuned, it could give rise to bioresins and hydrogels with broadly different characteristics, printability, and biological performances. In this study, we investigate the design of bioresins obtained from gelatins with varying molecular weight (MW) and Degree of Modification (DoM) to produce a library of materials compatible with a multi‐wavelength VBP printer and to identify groups of bioresins able to act as suspension baths printing and for EmVP. The effect of the synergy between MW, DoM, and temperature on the mechanical and rheological properties, and on the printability via VBP is studied. Bioresins suitable as mechanical support and as soft viscoelastic matrices for the culture of pancreatic cells are identified. Notably, a subset of gelatins and GelMA that exhibit shear‐yielding behavior in a stable manner over multiple hours was discovered, overcoming the limits of currently utilized gelatins, and offering a new material as ready‐to‐use suspension media for in‐bath printing, with improved resolution over microgel‐based methods. Finally, leveraging a multi‐color optics VBP printer, well‐aligned multi‐material and multi‐cellular, complex geometries were produced and demonstrated, by means of sequential VBP and EmVP. We expect that the ease of applicability of this method will extend the accessibility of volumetric printing for producing advanced, heterocellular tissue models for applications in tissue engineering and regenerative medicine.

## 2 Results and Discussion

### 2.1 An “Old” Polymer Under a New Light: Generating A Library Of Gelatin‐Based Hydrogels For VBP With Tunable Printability and Mechanics

**Figure 1:**
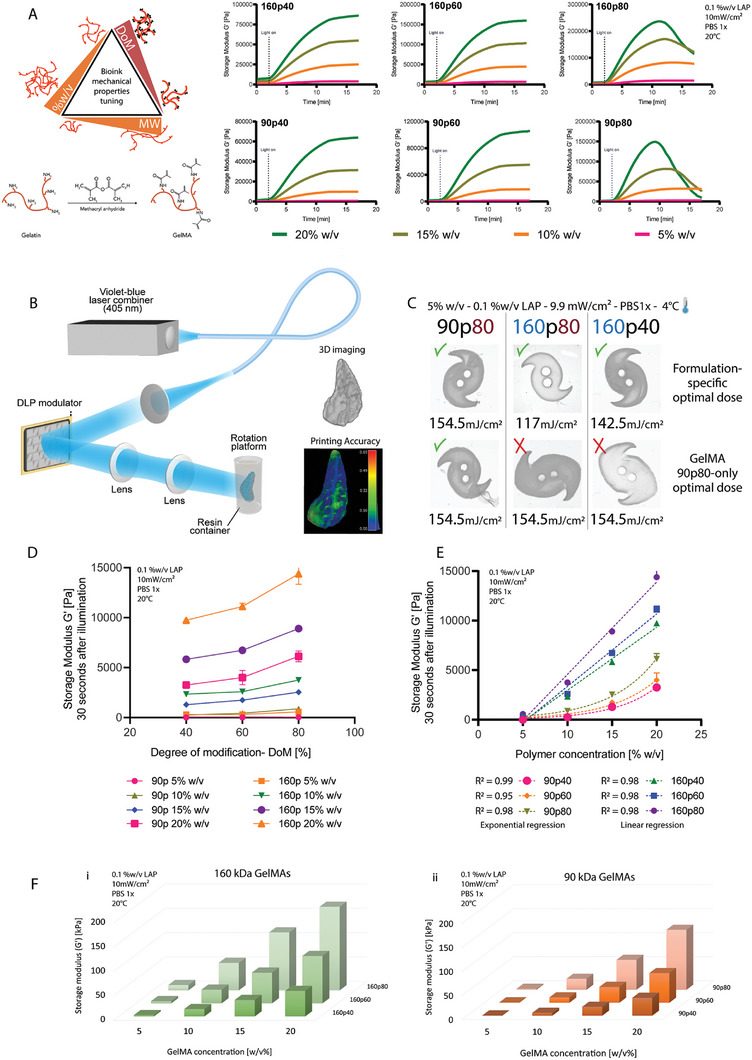
Library of the mechanical properties of the different GelMA hydrogel formulations and how they affect volumetric printing parameters. A) Schematic representation of the GelMA reaction process and overview of the different parameters involved in the design and tuning of the final bioink or bioresin mechanical properties. Representative (*n =* 3) photo‐rheology curves for the 90 kDa and the 160 kDa GelMAs with different degrees of modification (40%, 60%, and 80%), at different polymer concentrations (5% w/v, 10% w/v, 15% w/v, and 20% w/v). B) Experimental setup for computational axial lithography, showing a volumetric printed pancreas model. C) Printing light dose comparison between optimal values specific for the different GelMA formulations (5%w/v), and a reference value (90p80 optimal light dose). D) The hydrogel strengths of the various GelMA formulations at different concentrations (values of *G*’ after 30 seconds of exposure). An increased degree of modification leads to greater hydrogel strength. E) The relationship between hydrogel storage modulus and concentration is different depending on the average molecular weight (values of *G*’ after 30 seconds of exposure). For the 160 kDa GelMA, with increasing concentration, the storage modulus scales follow a linear trend. Conversely, at lower MW (90 kDa), storage modulus scales follow a power law, as a function of the concentration (*n =* 3). F) 3D overview of the storage moduli (*G*’) distribution of the GelMA hydrogels with 160 kDa MW i) and 90 kDa MW, as determined through photo‐rheology (*n =* 3).

In VBP, printing accuracy relies primarily on the ability to precisely deliver spatially controlled light doses with the printing volume, and on the kinetics of cross–linking of the bioresin. The latter is naturally dependent not only on the light dose and photoinitiator concentration but also on the available photoreactive groups (methacrylates, in the case of GelMA), which is a function of the molecular weight and degree of modification. Therefore, starting from type A porcine GelMA formulations that have average molecular weights of 90 and 160 kDa, with various degrees of modification (40, 60, or 80%), we generated a library of mechanical properties for the different GelMA hydrogels formulations (name coding XpY refers as: X = MW, p = porcine and Y = DoM). **Figure** **1**A depicts the photo‐rheological data of GelMAs with a molar mass of 90 kDa and 160 kDa at DoM of 40, 60, and 80%, and at concentrations of 5, 10, 15, and 20% w/v, in the presence of lithium phenyl‐2,4,6‐trimethylbenzoylphosphinate (LAP) as photoinitiator. Regardless of the MW, all formulations rapidly Cross–linked and reached a plateau and final storage modulus within 10 min of illumination (*G*’ values reported in Table S1, Supporting Information). The formulations that were expected to produce the densest hydrogel networks (concentration >15%, 80% DoM) displayed syneresis, the partial shrinking of the hydrogel induced by expelling water not bound to the tight polymeric mesh. This effect is visualized in the rheometer by an artefactual drop in storage modulus, caused by a loss of contact between the gel and the rheometer geometry upon shrinking.^[^ ^16^, ^17^ ^]^ To counter this artifact and still have a representative characterization of their high mechanical properties, a compression test was performed on GelMA 160p80 and 90p80 at 15% and 20% w/v (Figure S1, Supporting Information). Compression modulus values of 63.12 ± 3.2  and 120.85 ± 16.1 kPa were measured for GelMA 90p80 at 15% w/v and 20% w/v, respectively, and values of 105.2 ± 21 and 174.42 ± 0.9. This data supported how those formulations resulted in highly stiff and did not soften during the photo‐exposure step. The cross–linking kinetics of GelMA‐based hydrogels play a pivotal role during light‐based fabrication processes, as it affects the final mechanical properties and the printing resolution of the final model. As well established in the field of photoresponsive hydrogels, increasing the irradiation intensity, would also lead to a faster progression of the gelation, and therefore, result in higher storage modulus values compared to gels exposed to lower light intensities, if the irradiation time is kept constant, and the measurement is performed before the plateau of gelation is reached, as we also confirmed with experimental data (Figure S2, Supporting Information). Overall, characterizing the light dose needed to reach an optimal cross–linking condition is especially crucial when such hydrogels are employed for volumetric bioprinting. With this technique, since the whole bioresin volume is continuously photoexposed, excessive printing time can lead to off‐target polymerization and loss of printing fidelity.^[^ ^1^, ^2^, ^3^ ^]^ For this reason, we characterized how the interplay between the molar mass and degree of modification translated to the optimal printing dose within our volumetric printing set up, a schematic of which is represented in Figure 1B, together with a proof‐of‐concept 3D print of a pancreas model. In Figure 1C GelMAs 160p80, 160p40, and 90p80 were selected to provide a comparison between a) formulations with the same MW, but different DoM (160p80 vs 160p40), and b) formulations with the same DoM, but different MW (160p80 vs 90p80). All formulations were printed at 4 °C after thermal gelation occurred placing the printing vial on ice. The top row shows the results of prints performed at light doses optimized for each formulation, so that the printed constructs correctly display the same feature dimensions as the original.stl file, resulting in 154.5, 117, and 142.5 mJ cm^−2^ for GelMA 90p80, 160p80, and 160p40, respectively. These values correspond to a printing time of 16 s, 12 s, and 14 s. The data underlines how the higher the MW, the lower the required optimal light dose, and the higher the DoM, the lower the optimal light dose. Conversely, the bottom row shows the results of printing those same formulations at a fixed light that is optimal for only GelMA 90p80 (154.5 mJ cm^−2^) but revealed to be not ideal for the remaining two resin formulations (which led to overcured prints). These results underlined how the optimal printing light dose is indeed unique for each MW and DoM combination.

Besides reaching high printing accuracy, an optimal printing dose resulted in specific mechanical properties of the final Cross–linked constructs. This could be observed by comparing the storage modulus values of the different formulations at the 2.5 min mark (after 30 seconds of illumination in the rheometer), which is the time needed to deliver a light dose in the range of the one delivered by the volumetric printer (few tens of seconds). The data shown in Figure 1D confirms that with increasing DoM the storage modulus of the GelMA hydrogels increases too, in line with previous studies on GelMA‐based hydrogels.^[^ ^18^ ^]^ Similarly, with increasing GelMA concentrations (w/v%) the storage modulus of the hydrogel will increase.

Figure 1E shows how for the 90 kDa GelMAs a power‐correlation was established, with *R* ^2^ = 0.99, 0.95, and 0.98 respectively for GelMA 90p40, 90p60, and 90p80. The 160 kDa GelMAs showed, interestingly, a linear type of correlation R^2^ = 0.98 for all the 160 kDa GelMAs, with higher molar mass showing increased stiffness. This trend can also be observed at a glance, with the 3D representation of the dataset reported in Figure 1F, with the softest gel, 90p40 at 5%, showing a storage modulus as low as 379±47 Pa, and the stiffest gel, 160p80 at 20% showing values of 171.46 kPa. Hence, from these datasets, it can be appreciated how 90p60 and 160p40 GelMAs at 15% w/v can potentially have similar stiffness values, thus opening opportunities for future mechanistic studies in which cell behavior can be studied in gels with invariant stiffness, but tunable mesh size.

Finally, to maximize the consistency and reproducibility of the hydrogel formulations, it is also important to consider the effect of photo‐initiator and salt concentration. On the one hand, specifically for VBP, higher initiator content also affects light attenuation within the vial, therefore requiring to adjust the incident light intensity to achieve cross–linking, as previously reported.^[^ ^3^ ^]^ Of particular relevance for processing hydrogels with varying MW, DoM, and polymer concentration, the content of photoinitiator affects the photocross–linking kinetics, as higher concentrations will lead to faster generation of reactive species upon photoexposure. Moreover, since each hydrogel formulation displays a different amount of reactive groups available for the acrylate kinetic chains to form, maintaining a constant LAP concentration across each experimental group contributes to the differences in gelation kinetics and the amount of excess radicals that can potentially damage embedded cells. While from a practical point of view, this difference can be accounted for by adjusting the light dose during printing, strategies that optimize the LAP concentration as a function of the available methacrylate moieties can also be envisioned. A dissertation on how varying the photoinitiator concentration affects the curing of hydrogels with different DoM and MW has been included in the Supplementary Information (Figures S3 and S4, Supporting Information). Lastly, the ionic content of the aqueous buffer used to produce the bioresin also influences both the gelation kinetics and ultimate gel stiffness, with lower stiffness shown for increasing concentrations of phosphate buffer saline (PBS) in the pre‐polymer solution (Figure S5, Supporting Information). For the experiments performed in the current and following sections, PBS 1X was selected as a medium to dissolve the GelMA polymers.

### 2.2 GelMA Processed at Different Temperatures Leads to Different Strength, Cross–linking Rates and Printing Time

**Figure 2:**
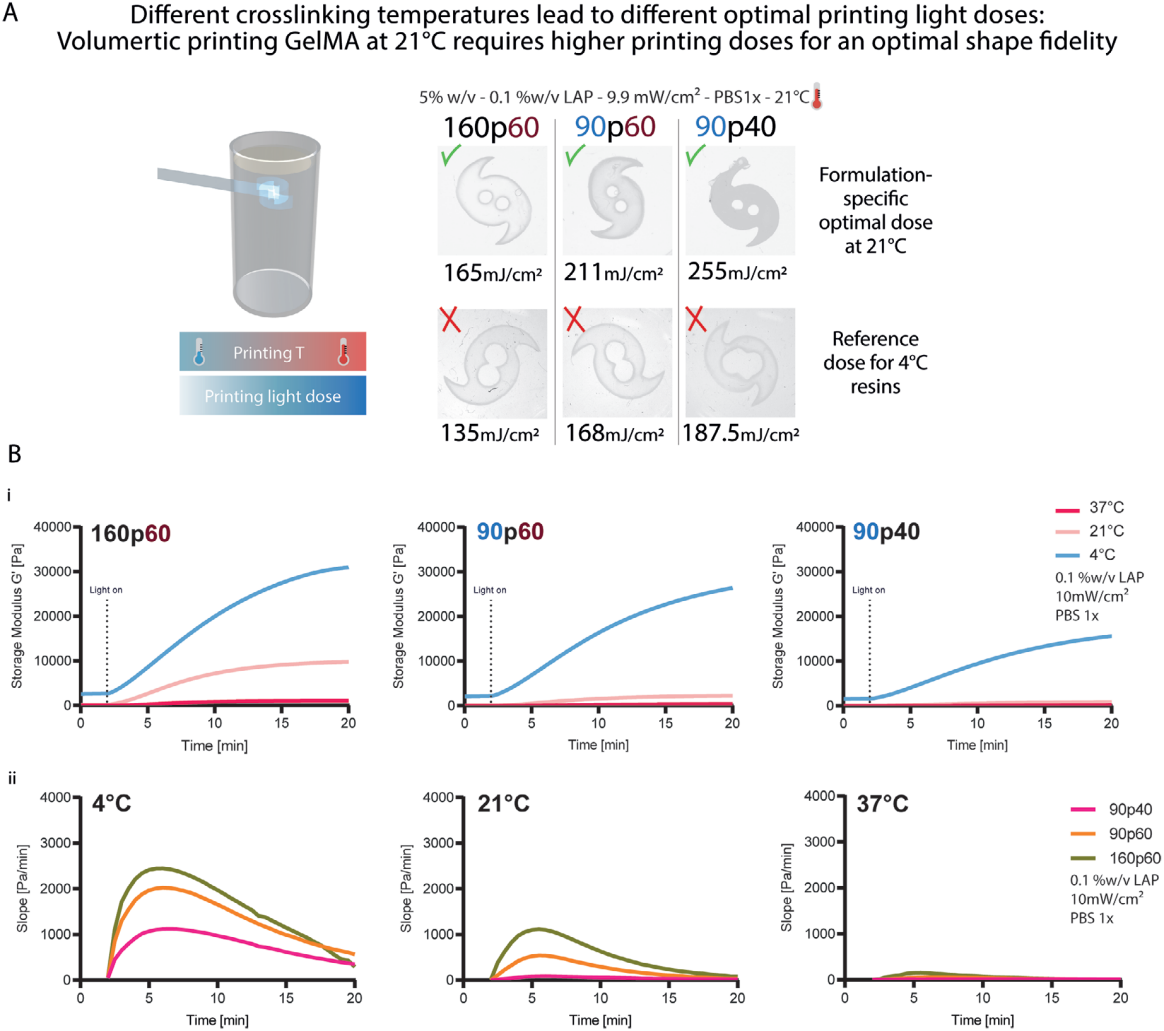
Effect of temperature on volumetric printing parameters, on the final mechanical properties and cross–linking kinetics of different GelMA hydrogels. A) Higher cross–linking temperatures lead to higher optimal printing light doses. Printing light dose comparison between optimal values specific for the different GelMA formulations (5%w/v) printed at room temperature, and a reference value (optimal values specific for the different GelMA formulations printed at 4 °C). B) i) Storage moduli (*G*’) of 160p60, 90p60, and 90p40 at 5% w/v at 4 °C, 21 °C and 37° (*n =* 3), showing that photo‐curing at decreased temperature results in increased hydrogel stiffness. ii) Photo‐cross–linking rates represented by the first derivatives of the photo‐rheology curves of 160p60, 90p60, and 90p40 at 5% w/v at 4 °C, 21 °C and 37° (*n =* 3), showing how with increasing temperature, the curing rates slow down. Photorheology was performed with 10 mW cm^−2^, 300 mJ cm^−2^ light sources.

Within the volumetric printing process of GelMA, the thermoreversible behavior of gelatin is particularly advantageous, as bioresin‐filled vials are placed at cold temperatures to promote thermal gelation, which prevents cell sedimentation.^[^ ^3^, ^19^ ^]^ We therefore characterized how the cross–linking temperature could affect the curing kinetics and the optimal printing dose within our volumetric printing set up (**Figure** **2**). For Figure 2A, a similar approach to Figure 1C was adopted, using GelMAs 160p60, 90p60, and 90p40, to provide a comparison between a) formulations with the same MW, but different DoM (90p60 vs 90p40), and b) formulations with the same DoM, but different MW (160p60 vs 90p60), focusing on highlighting the relationship between the printing temperature and the optimal light dose. All formulations were printed at 21 °C measured after thermal gelation occurred at room temperature overnight. The top row shows the results after finding the optimal printing light dose for each formulation, resulting in 165, 221, and 255 mJ cm^−2^ for GelMA 160p60, 90p60, and 90p40, respectively. These values reflected the same trend observed in Figure 1C: when comparing materials with similar DoM, a lower optimal light dose is needed when the material has a higher molecular weight.

The bottom row, instead, shows the results of printing each resin formulation at the optimal light dose needed if the sample had been set to 4 °C (135, 168, and 187.5 mJ cm^−2^ for GelMA 160p60, 90p60, and 90p40, respectively), even though the resins are actually printed at 21 °C. In this case, all printed samples were under‐polymerized. This is in line with what is shown in the photorheology curves (reported in Figure 2B), which show faster cross–linking (thus requiring lower light doses) at lower temperatures. Further, the results reported in Figure 2A exemplify how each combination of temperature, MW, and DoM is also characterized by a unique optimal printing dose.^[^ ^19^, ^20^ ^]^ Printing at room temperature using optimal doses for the 4 °C conditions led to under‐polymerized structures, and therefore failure to print.

To exemplify the thermo‐sensitivity of GelMA in hydrogel production, an experiment was designed using 90p40, 90p60, and 160p60, all at a concentration of 5% w/v in 1x PBS. The various GelMA resins were photo‐cured and a photo‐rheometer was used to showcase the temperature effects in hydrogel cross–linking (Figure 2B). The temperature at which the GelMA resins were cured were 4 °C, 21 °C (room temperature), and 37 °C.

In line with the literature, the photo‐rheological data presented in Figure 2Bi showed how the hydrogel stiffness, as represented by the storage modulus (*G*’), is greatly affected by the temperature at which the GelMA is being cured into a biopolymeric network.^[^ ^21^, ^22^ ^]^ The cross–linking rates, represented by the first derivatives of the photo‐rheology curves, are temperature‐dependent as well, translating into faster rates at decreased temperature (Figure 2Bii). In a typical cell printing experiment, the preparation of the bioresin and cell suspension in the prepolymer solution is carried out at room temperature (21 °C). Next, regarding volumetric printing, in which the GelMA is cooled to induce physical gelation, the curing kinetics and hydrogel are further affected by the temperature (4 °C).^[^ ^23^ ^]^

Finally, to get a qualitative valuation of the amount of Cross–linked polymer chains as a function of the cross–linking temperature, a sol‐gel analysis was performed and the sol‐fraction % was calculated for one of the GelMA formulations, Cross–linked at different temperatures (e.g., 4 °C and 21 °C).

Results of the sol‐fraction % for GelMA 90p60 Cross–linked at different temperatures (Figure S6, Supporting Information) showed how a significantly lower sol‐fraction % is measured on samples Cross–linked at 4 °C (7%), compared to Cross–linked samples at 21 °C (10.8%). This could be attributed to a higher (faster) conversion of MA when GelMA 90p60 was cross–linking at lower temperatures. Such is likely due to the higher entanglement of the polymer chains at lower temperatures (being GelMA a thermoresponsive polymer, which physically stabilizes via gelation at low temperatures).

### 2.3 Leveraging Low MW GelMA to Match Soft Tissues Microenvironment Mechanical Properties

**Figure 3:**
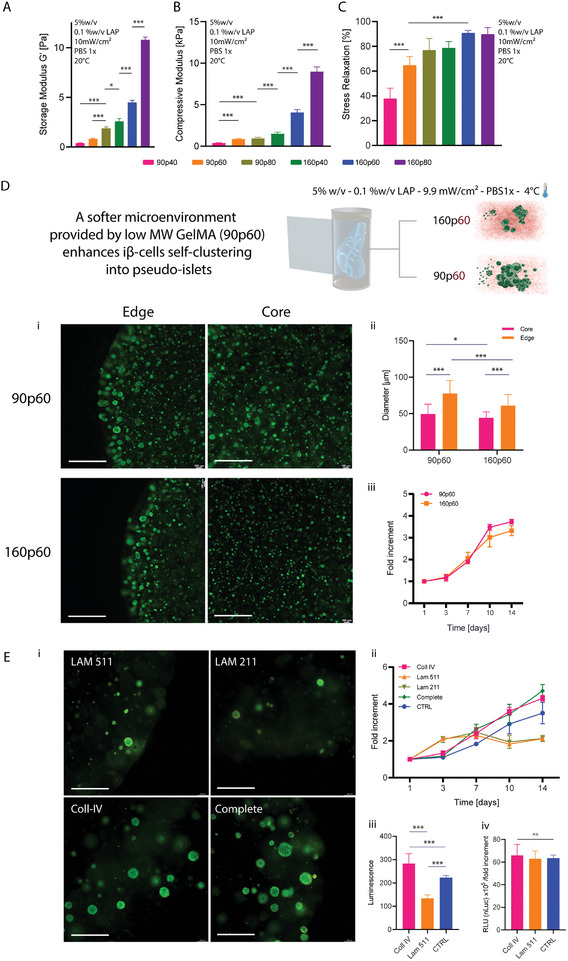
Optimizing 3D cell culture environments via tuning of the GelMA properties via molecular weight and degree of functionalization modulation and targeting the mechanical profile of soft tissues. A) storage moduli at the 7 min mark (*G*′) B) Compressive mechanical properties and C) the stress relaxation % after 2 min for the different GelMA formulations at 5% w/v concentration (*n =* 4). D) Recapitulating an optimal environment for the iβ‐cell line leveraging 90p60 GelMA mechanical properties. i) Effect of molecular weight of GelMA bioresins (90p60 and 160p60) at 5% w/v concentration (1%w/v LAP, dissolved in PBS 1X) on ii) ability to proliferate and form islet‐like clusters iβ‐cell line and iii) metabolic activity (*n =* 4). Scale bars: 500 µm E) i) Effect of selected salient macromolecules from the pancreatic ECM on ii) metabolic activity, iii) overall, and iv) fold‐increment normalized luciferase secretion of iβ‐cell line volumetric printed in GelMA 90p60 (*n =* 4). Scale bar: 300 µm.

Having elucidated how GelMA‐based materials result in different final properties depending on the polymer characteristics and on which the bioprinting process is adopted, the biological performance of different GelMA from the previously created library was investigated (**Figure** **3**). As proof‐of‐principle, we aimed for an application in the field of endocrine pancreatic tissue engineering. For this purpose, we bioprinted constructs laden with iβ‐cells, an engineered cell line genetically engineered to produce and store insulin in intracellular vesicles (together with the bioluminescent reporter nanoLuc) and to rapidly release it on demand as a response to visible light exposure (475 nm). This enables on‐demand extracellular delivery of the cargo within the vesicles.^[^ ^24^, ^25^ ^]^ To select hydrogels within the GelMA library produced with the previous experiments that match the mechanical properties of soft tissues, we focused on GelMA hydrogels at the lowest polymer concentration (5% w/v), and we further investigated their compressive stiffness, and viscoelastic behavior via a stress‐relaxation assay. Figure 3A shows how the shear storage modulus (measured by rheometry) increases for higher molar mass and DoM, and this trend is reflected also in the compressive modulus, as measured in a quasi‐static uniaxial compression assay via dynamic mechanical analysis (DMA) (Figure 3B), consistent with the literature.^[^ ^21^, ^26^ ^]^ More specifically, modulus values of 5% w/v GelMAs (0.1% w/v LAP concentration) ranged from 0.4 ± 0.04 to 0.96 ± 0.1 kPa for the samples 90p40 and 90p80, respectively, while ranged from 1.5 ± 0.18  to 8.9 ± 0.58 kPa for the samples 160p40 and 160p80.

Notably, working with low MW gelatin allowed us to modulate the viscoelastic behavior of the hydrogels. From the stress relaxation curves, the stress relaxation % (ratio between peak stress, and stress at a plateau after relaxation) was extracted (Figure 3C). While high MW gelMA showed a mainly elastic behavior with slow relaxation (index 89.8% for GelMA 160p80), decreasing MW and DoM allowed to gradual introduce faster relaxation (index 37.8% for 90p40), in a range suitable to mimic several native tissues. To compare the compressive and viscoelastic mechanical properties of the GelMA formulations to soft tissues, we therefore also performed measurements on a porcine pancreatic tissue via mechanical indentation. The compressive properties of the porcine pancreas are shown in Figure S7 (Supporting Information), over indentation across multiple areas of the tissue. The compressive modulus ranged from 2.3 to 87 kPa, while for the stress relaxation, the average stress relaxation % ranged from 7 to 34%, after 30 seconds of relaxation, ranges comparable with the lower DoM and MW GelMAs.

An initial biological screening was performed by casting iβ‐cells in all the GelMA formulations at 5% w/v and viability was qualitatively tested over 7 days of culture via Live/Dead assay (Figure S8, Supporting Information). A basal membrane extract analog to Matrigel (commercially available under the name Geltrex) was tested as a control. The GelMA 90p40 was unstable in culture and degraded within the first day of the experiments. For all other samples, from Live/Dead images, cells appeared highly viable both after encapsulation and after 7 days of culture, being homogenously distributed throughout the volume of the hydrogels. The gelMA samples also provided improved stability over degradation compared to the basal membrane extract, which fully degraded by day 7. Considering these results, the 90p60 formulation was selected for further screening on VBP‐bioprinted samples, as this formulation displayed low‐stress relaxation %, and shear modulus and compressive stiffness comparable to the native pancreas.^[^ ^27^, ^28^ ^]^ To reveal the effect of molecular weight on pancreatic cell proliferation and the ability to form clusters of pseudo‐islets, which is necessary for these cells to functionally secrete insulin,^[^ ^9^, ^29^ ^]^ 160p60 GelMA was also selected to be compared to 90p60, keeping fixed the degree of modification. Cell metabolic activity and self‐clustering capability were therefore tested by comparing volumetric printed gels at 5% w/v, 0.1% w/v LAP embedding 1 × 10^6^ iβ‐cells mL^−1^.

The influence of molecular weight on pseudo‐islet cluster formation is shown in Figure 3D. Live/Dead images (Figure 3Di) and quantitative measurements showed how a softer microenvironment enhanced the cell clustering and cluster growth, resulting in significantly larger pseudo‐islets in GelMA 90p60, compared to 160p60 (Figure 3Dii). This might be attributed to the lower stiffness and faster relaxation kinetics of GelMA 90p60, due to the shorter polymer chains, which result in loose and accessible for cells to self‐organize.^[^ ^30^ ^]^ Moreover, we found pseudo‐islets with bigger diameters closer to the edges of the gels in both formulations, likely due to the shorter diffusion distance for nutrients.^[^ ^31^ ^]^ The overall suitability for cell culture of the hydrogels was quantitatively confirmed by increased metabolic activity values over a period of 14 days, with a 3.7 and 3.3 fold increment for GelMA 90p60 and 160p60, respectively (Figure 3Diii). Having identified the 90p60 formulation as preferred for iβ‐cell culture, we further investigated how the addition of ECM components, which are known to be presented in the pancreatic microenvironment,^[^ ^32^, ^33^, ^34^, ^35^, ^36^ ^]^ could affect long‐term cell proliferation and cluster formation (Figure 3E). In particular, collagen type IV, laminin 511, and laminin 211 were chosen because of their well‐known contribution to the regulation of islet morphology and survival.^[^ ^33^, ^37^, ^38^, ^39^ ^]^ iβ‐cells were volumetrically printed in GelMA 90p60 enriched with the single ECM components or with a mix of the three proteins (termed “complete” formulation), and cultured for 14 days. GelMA 90p60 without any additive was used as a control. Live/Dead images reported in Figure 3Ei showed overall high viability and self‐clustering behavior in all the formulations, with a qualitatively higher presence of pseudo‐islets in the collagen IV and complete formulations, but not in the laminins‐only supplemented samples. Further a higher metabolic activity on day 14 for the conditions with collagen IV and the combination of all ECM components was measured (Figure 3Eii), with a fold increment respectively of 4.3 and 4.7 times. Statistically significant differences were found between controls and the collagen IV and complete formulations on days 3, 7, 10, and 14, and between the collagen IV and complete groups versus the laminins‐supplemented conditions on days 10 and 14. These data indicate that the addition of collagen IV is sufficient to enhance iβ‐cell proliferation and islet formation, whereas laminin 511 and 211 appeared to reduce proliferation. Finally, to test if the functionalization translates also to improved insulin secretion, we performed a functional assay, with the pristine GelMA controls, and samples enriched with collagen IV and laminin 511. The amount of insulin released by the pseudo‐islets was indirectly assessed by measuring the bioluminescent reporter nanoLuc. Results (Figure 3Eiii) showed a significantly higher reporter concentration in the culture medium collected at day 14 in the collagen IV formulation, compared to the control (only GelMA 90p60) and the laminin 511 (resulted as the formulation with the lowest fold increment in metabolic activity). Our findings indicate that this beneficial effect is primarily driven by the improved cell proliferation in the presence of collagen IV, rather than in the boosting of the capability of individual cells to produce insulin, as confirmed by the data normalized against the total cell number (Figure 3Eiv). Finally, while our analysis confirms that the 90p60 formulation is suitable to promote the growth and clustering of endocrine cells, we also provided a proof‐of‐concept indication of the suitability of using low molecular weight GelMAs for supporting the culture of vascular cells, needed for mimicking the vascularized stroma (Figure S9, Supporting Information). Therefore, a co‐culture of Human Umbilical Vein Endothelial Cells (HUVECs, GFP‐tagged, 2.5 × 10^6^ cells mL^−1^), with 5 × 10^6^ hMSCs mL^−1^ was photo‐encapsulated in a GelMA 90p60 and 160p60 at 5 w/v% concentration. Capillary networks were successfully formed at day 7 only in GelMA 90p60 (Figure S9Ai–v,B, Supporting Information), compared to the 160p60 formulation (Figure S9Ci–iii, Supporting Information), further confirming how lower molecular weight gelatins provide a more permissive environment for cellular self‐assembly across different types of cell lines and applications. It should be noted that no perfusion or interstitial pressure gradients were applied in this time frame. Since these parameters are known to be necessary for promoting capillary vessel maturation and are known to support vascular lumen opening and long‐term stability in culture,^[^ ^40^, ^41^ ^]^ future and longer‐term studies focusing on vasculogenic potential could include dynamic culture settings.

### 2.4 Anisotropic and Multi‐Cellular GeMA‐Based Constructs Via Multi‐Material Volumetric Bioprinting

**Figure 4:**
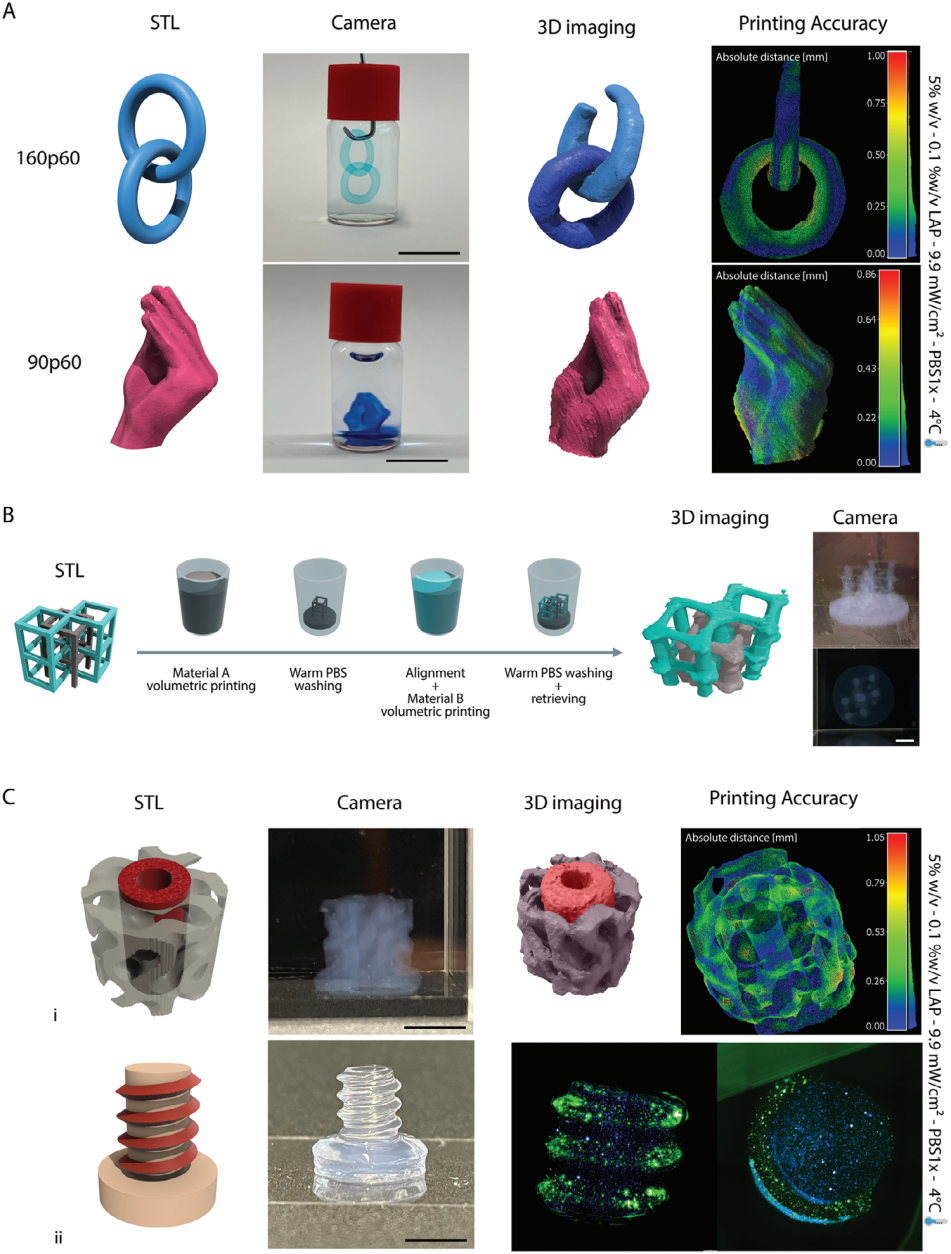
Sequential multi‐material volumetric printing. A) Examples of complex structures obtained via volumetric bioprinting of GelMA formulations with different molecular weights, including two interlocked rings, and a model of a hand with pinched fingers. Quantitative printing accuracy 3D maps are provided as a difference between the original STL file and the printed object, showing local variations in the size of the printed features across the whole volume of the objects. Scale bars = 10 mm. B) Graphical overview of the sequential multi‐material volumetric printing process, and demonstration of two printed independent and intertwined hollow cube scaffolds (digitally colored in grey and cyan in the 3D light sheet images to facilitate visualization). Scale bar = 3 mm C) Example of multi‐material prints: i) a dual material printed reinforced gyroid (Scale bar = 4 mm) and ii) a dual material printed screw (Scale bar = 5 mm) with a core and thread printed with formulations embedding iβ‐cells with different stainings (DiL and Did. Vybrant Multicolor Cell‐Labeling Kit, Thermo Fischer Scientific, The Netherlands).

After successfully demonstrating the ability to photo‐Cross–link the different GelMA formulations, and showing how GelMA 90p60 could be sculpted via volumetric bioprinting while creating a permissive environment for cell culture, we tested the possibility of forming multi‐material constructs containing soft materials for cell embedding and stiffer ones to provide mechanical stability over the culture period. To achieve this, we first set up a strategy to volumetrically print multiple GelMA‐based materials in a sequential fashion. In order to create models based on GelMAs with different mechanical characteristics, we selected GelMA 160p60 formulation to be sequentially printed with GelMA 90p60 (**Figure** **4**). The design freedom provided by the volumetric bioprinting process allowed the production of models with movable or articulating parts, like the intertwined rigs printed with GelMA 160p60, or complex self‐standing models, like the one resembling the pinched fingers “Italian hand” model emoticon, printed with GelMA 90p60 (Figure 4A). To finally test the printing accuracy of the fabricated structures, we performed a detailed quantitative analysis by calculating the differences between the original STL files and the printed model volumes (Figure S10, Supporting Information). The color code in the heat maps indicates the dimensional deviation from the STL in mm, as also shown on the scale on the right side of the images (C2C absolute distance). For the intertwined rigs, the average deviation from the original STL was 0.28 ± 0.20 mm, while for the Italian hand 0.20 ± 0.14 mm. Having tested the volumetric printability of both GelMA formulations, multi‐material printing was subsequently performed via a sequential approach (Figure 4B).

A first volumetric print was performed, then the unpolymerized resin was washed out, and the vial containing the structure printed with the first material was filled with the second material. The alignment of the projections of the model meant to be overprinted with the previously printed structure was performed by leveraging wavelengths (in our case 520 nm) that are orthogonal to the photocross–linking, which is at 405 nm. This is crucial to prevent unwanted cross–linking, with a subsequent low printing accuracy, if the aligning process was conducted with the same wavelength used to Cross–link the bioresin. The second and, eventually, final volumetric printing step was therefore performed.

This approach allowed to printing well well‐aligned, and cell‐laden objects. For example, in Figure 4Ci, we showed the combination of 160p60 and 90p60 by sequentially printing a reinforced gyroid. The 160p60‐based cylindrical scaffold at the core of the 90p60‐based gyroid is a possible strategy to exploit the stronger mechanical characteristics of GelMA 160p60 by providing additional stability to the softer GelMA 90p60 structure (Figure S11, Supporting Information). Even in this case, the printing accuracy analysis showed promising values, with an average deviation from the original STL of 0.25 ± 0.19 mm.

Moreover, to prove the possibility of printing multi‐cellular constructs on top of anisotropic models, a pancreatic beta‐like cell line (iβ‐cell line)^[^ ^24^ ^]^ was stained with different fluorescent membrane dyes (DiL and Did. Vybrant Multicolor Cell‐Labeling Kit, Thermo Fischer Scientific) to facilitate visualization, and loaded in distinct GelMA 90p60 solutions.

We showed a sequentially volumetric printed screw, characterized by a 90p60‐based core and thread, seeded with the different iβ‐cell batches (stained with different fluorescent membrane dyes) (Figure 4Cii). The average deviation from the original screw STL was 0.25±0.24 mm (Figure S10D, Supporting Information). The herein‐presented comparison method was not covered before by any other research study for cm^3^‐scale complex structures based on soft hydrogels. Figure S10 (Supporting Information) showed a low dimensional error, less a 2%, similar to other studies carried out.^[^ ^42^, ^43^ ^]^ However, in our study as soft hydrogels were used, it should be kept in mind that these structures tend to swell post‐printing, therefore introducing a source of dimensional changes prior to the 3D imaging analysis.^[^ ^44^ ^]^ Overall, these experiments showed that multi‐material VBP utilizing materials with different mechanical properties is possible and allows the generation of both cell‐free and cell‐laden structures. Having proven this potential, paving the way to the next steps investigating how the unique properties of low molecular weight gelatins can be leveraged to enable new high‐resolution multi‐modal bioprinting techniques converging extrusion and light‐based biofabrication.

### 2.5 Low Molecular Weight Gelatins Formulations Enable Facile, Plug‐And‐Play Embedded Extrusion Volumetric Printing Due to Their Intrinsic Self‐Healing‐Like Behavior

Embedded extrusion bioprinting is a well‐established technique in the biofabrication field^[^ ^45^, ^46^ ^]^ leveraging an ideal support bath for extruding low‐viscosity materials.^[^ ^46^, ^47^, ^48^ ^]^ In traditional extrusion bioprinting, several artifacts are caused on the overhanging features due to gravity and surface tension‐driven effects.^[^ ^49^ ^]^ To overcome this problem, embedded extrusion printing leverages printing inside of a suspension bath, managing to keep the extruded structure in its predesigned 3D asset and preventing it from collapsing. However, the fabrication time increases cubically with the scaling factor, as for all extrusion‐based printing techniques, resulting in a disadvantage for cell‐laden constructs fabricated at the centimeter‐scale.^[^ ^3^ ^]^

**Figure 5:**
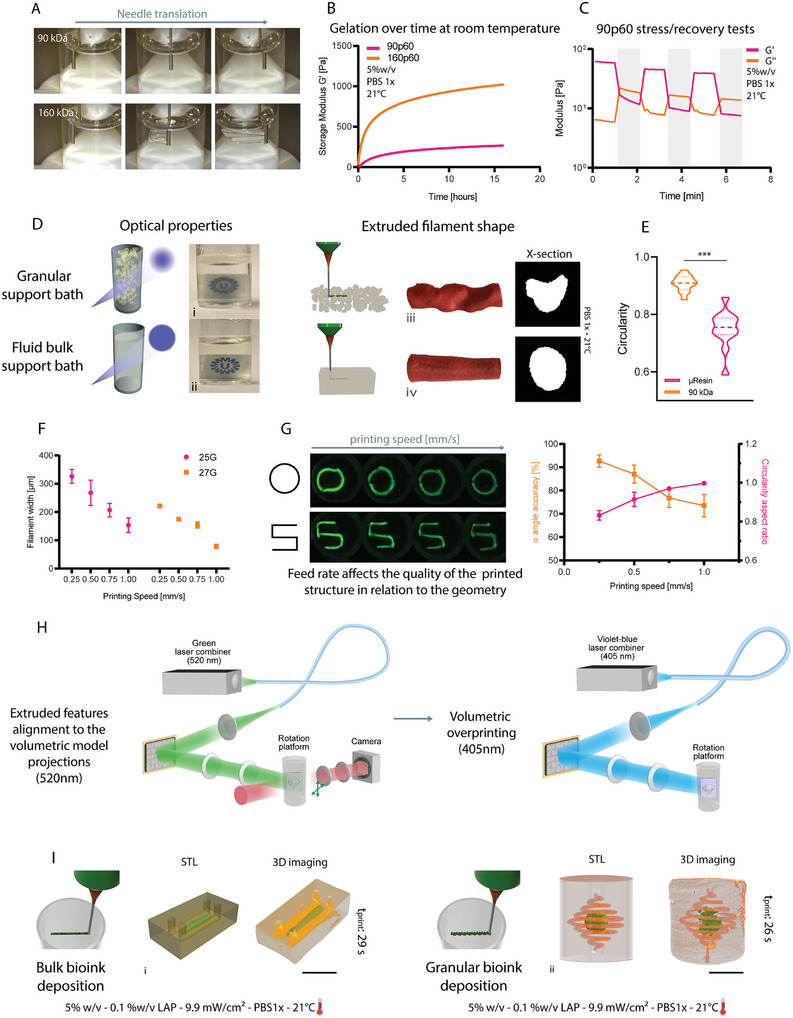
Low molecular weight gelatins enable seamless and omnidirectional extrusion printing as suspension baths, and Embedded extrusion Volumetric Printing (EmVP). A) Fluid bulk support baths made of 90 kDa gelatin and/or GelMA (see Figure S9) show self‐healing‐like properties, whereas 160 kDa GelMA‐based baths show slow recovery and mechanical damage caused by the movement of an immersed extrusion nozzle. B) Gelation over time at room temperature of GelMA 90p60 and 160p60. C) 90p60 GelMA rheological properties make it suitable as a suspension bath for embedded extrusion bioprinting. Self‐healing through low (unshaded, 1% strain, 1 Hz) and high (shaded, 500% strain, 1 Hz) strain cycles (*n =* 3). D) Comparison between μResin and 90p60‐based support baths: qualitative characterization of the optical properties (light scattering) of the microparticle‐based bioresin i) and of the fluid bulk support bath ii). Qualitative characterization via 3D reconstruction and cross‐section visualization of the embedded filaments extruded in the microparticle‐based bioresin iii) and in the fluid bulk support bath iv). E) Quantitative characterization of the circularity of the cross‐section from the embedded filaments (*n =* 12). F) Printing accuracy of the two formulations, calculated by measuring the filament width as a function of the nozzle diameter and printhead translational velocity (*n =* 5). G) Angle accuracy and circularity aspect ratio quantification at different printing speeds of the embedded extruded filament in 90p60 fluid bulk support bath (*n =* 4). H) Multi‐wavelength approach for calibration and printing during EmVP. 520 nm green light, far from the LAP excitation spectrum, is used for the manual alignment of the vial in the Z axis and XY plane, in order to match the position of the extruded features with the initial angle at which the vial will start to rotate and send, in synchrony, the projections of the object to be overprinted. Subsequently, the volumetric printing process starts using a violet‐blue 405 nm laser line. I) Schematic representation of the EmVP process showing the fabrication of a microfluidic chip (channels diameter: 1 mm) i) via bulk bioink extrusion and centimeter‐scale complex structure (channels diameter: 500 µm) ii) via granular bioink extrusion. Scale bars = 4 mm.

The support bath in which constructs are suspended upon (bio)printing is often complex in formulation, requiring a mixture of multiple materials or lengthy processes and purification steps to obtain minute microgel baths, for example via polymer coacervation,^[^ ^50^ ^]^ which limits the throughput and broad accessibility of such promising techniques. Alternatively, suspension baths made from bulk polymer gels have been proposed, although these also typically require lengthy preparation steps,^[^ ^51^ ^]^ or the mixing of multiple components to obtain the proper viscosity^[^ ^10^ ^]^ and stress relaxation properties. These specifications are needed to allow the extrusion needle to pass through the viscous bath resin to deposit the ink‐material while maintaining the supporting and relax‐flow characteristics of the bath‐resin.^[^ ^52^, ^53^, ^54^, ^55^ ^]^ Our data, revealed that fluid bulk baths for embedded bioprinting can be produced using not only GelMA 90p60 but, also using any low MW gelatin formulations, independently of the DoM, as shown by using unmodified 90 KDa gelatin as a support bath, without the need of additive, harmful surfactants or rheology modifiers (**Figure** **5**). Our bath resin is produced using a 5% w/v concentration, upon thermal gelation. Once gelated, 90 kDa gelatin showed self‐healing‐like properties which allowed the needle to translate through the bath without creating any scratch or cavity which would affect the bioink deposition quality. The opposite behavior was observed in 160 kDa gelatin, where grooves were created after needle translation (Figure 5A). The same trend was finally observed in the respective GelMAs (90p60 and 160p60). This proved how the modification didn't affect the possibility of using the low molecular weight formulation as a support bath for embedded extrusion printing (Figure S12, Supporting Information).

Specifically, 90p60 GelMA is better performing for being used as a support bath as the gel stiffness of the physically gelated GelMA is considerably lower (≈200 Pa) than 160p60 GelMA (≈1000 Pa) after 12 h of gelation at room temperature (Figure 5B). Most notably, these advantageous properties are stable over multiple hours, facilitating bath storage and performing long sessions of experiments, in contrast with data reported for commonly used gelatins and GelMA, which display flow properties ideal for bioprinting only over a short window of time and temperature during their gelation kinetics.^[^ ^56^ ^]^

Additional characterization on the 90p60 GelMA was conducted to further study this behavior. Stress/recovery tests were performed by alternating low (1%) and high (500%) strain at 1 Hz frequency and results showed a higher G″, compared to *G*′, when the higher strain was applied (Figure 5C). This showed how 90p60 went through a solid‐liquid transition when a strain was applied, which could be representative of the needle translating through the gel during the extrusion phase, preventing, therefore, the gel from cracking. After the strain was removed, higher *G*′ values were registered compared to *G*″, showing a liquid‐solid transition of the gel which led to the possibility of supporting the extruded filament. In addition, a shear strain sweep test was performed, and results supported the previous data, showing a cross point of *G*′ and G″ at 480% strain, resulting in a solid‐liquid transition (Figure S13A, Supporting Information). Moreover, a shear thinning behavior was observed as the viscosity decreased as the shear strain increased and, under the same conditions, the shear stress increased in a nonlinear fashion (Figure S13B, Supporting Information). This behavior was observed both in non‐modified gelatin and modified (methacrylated) 90p60, thus opening the possibility to easily build suspension baths for bioprinting that can be either used as sacrificial, temporary support, but also covalently Cross–linked post‐extrusion printing. In particular, photo‐responsive suspension baths can be used for Embedded extrusion Volumetric Printing (EmVP), to sculpt the suspension bath into virtually any desired 3D geometry encasing extruded features, as previously demonstrated by our group using a granular bath made of GelMA microgels.

However, when the support bath is meant to be cross–linked via light‐triggered reactions, the optical properties play a crucial role as well. Compared to a granular one, a bulk bath resulted more transparent and permissive for light to cross the entire volume without having massive scattering effects, caused instead by the presence of gels microparticles in the granular formulations (Figure 5Di,ii). Moreover, the deposited filament within a granular bath showed a coarser and cracked shape, due to the presence of the microgels and their interstitial porosity that template the extruded filament.^[^ ^57^ ^]^ Additionally, using granular baths, also entails that, when extruding very low viscosity bioinks or cell suspensions, the bioink can partly filtrate into the inter‐particle porosity, compromising printing resolution.^[^ ^58^ ^]^ Conversely, a smooth and tight filament can deposited in the low molecular weight gelatin fluid bulk bath (Figure 5Diii,iv). In fact, measurements of the cross–sections of extruded filaments in different baths showed a higher circularity (90%) in strands deposited in fluid bulk support baths, compared to the granular one (74%) (Figure 5E), confirming higher shape fidelity of the filament, and thus to higher printing accuracy of the final extruded geometry. This new approach using low molecular weight single component baths allows therefore to easily obtain high resolution and shape fidelity, without the need for complex multi‐material blends or phase‐separation systems previously described in the literature to achieve such results.

Following the printability optimization, we managed to extrude features of 80 µm with a GG/PEGDA bioink, previously designed and characterized,^[^ ^9^ ^]^ which is a smaller dimension than the diameter of the smallest needle (27G) utilized in this study (Figure 5F).

Interestingly, the feed rate of the needle affects the quality of the printed structure in relation to the geometry. In particular, curved and round structures benefit from a higher printing speed, while squared structures and corners benefit from a slower printing speed. This relation was quantified by measuring the angle of squared structures and the circularity of round structures in relation to the printing speed (Figure 5G). This crucial aspect must be considered when considering extruding more complex structures comprising both rounded and sharp features, where the printing speed must be consequently adjusted in the g‐code in relation to the printed feature.

Finally, we used 90p60 as a fluid support bath to generate centimeter‐scale, geometrically complex constructs, such as microfluidic chips laden with precisely patterned multiple cell types or materials, by converging volumetric and extrusion bioprinting via EmVP, (Figure 5H). Briefly, the designed structure meant to be extruded and the structure to be subsequently volumetrically overprinted are designed as separated models. Embedded extrusion printing was initially performed directly in the volumetric printing vial. Then the vial was placed in a custom‐developed polychromatic volumetric printer. At this point, the multi‐wavelength setup of the volumetric printer is used to first align the extruded features with the projections from the DMD of the model supposed to be overprinted, and finally to perform the cross–linking. More specifically, 520 nm light is used for the alignment (Figure S14, Supporting Information), far from the excitation spectrum of LAP to avoid unwanted cross–linking, by sending the projections to the vial, followed by the manual adjustment to match the starting angle of the tomographic reconstruction process. Subsequently, the volumetric printing process was initiated using a 405 nm laser line where a series of light‐projections, delivered at a specific dose, recreated the pre/designed geometry onto the existing extruded feature. EmVP performed in fluid bulk support baths further enriches reported techniques by sculpting tens of seconds of different microfluidic geometries and complex models in a high throughput way. Finally, the vial containing the printed constructs was heated to 37 °C to melt the unpolymerized 90p60, and the sample was washed with prewarmed PBS. Figure 5Ii shows a biomimetic in vitro chip with a bioprinted strand and two parallel channels (channels diameter: 1 mm), potentially simulating blood and lymphatic vessel pair in a tumor chip.^[^ ^58^ ^]^ Moreover, Figure 5Iii shows the possibility of extruding in the fluid support bath not only bulk hydrogels but granular bioinks as well, creating microfluidic structures (channel diameter: 450 µm) with particulate and highly porous cores. All these multi‐materials complex structures were obtained through EmVP in less than a minute.

Finally, as a proof‐of‐concept of multicellular structure (Figure S15, Supporting Information), we first patterned iβ‐cells, embedded in a methylcellulose‐based bioink (already characterized in a recent work from our lab),^[^ ^9^ ^]^ into a GelMA 90p60 bath (5% w/v, 1% w/v LAP, dissolved in PBS 1X). The bath already contained homogenously suspended hMSC. Finally, the construct containing the two cell types was Cross–linked using the volumetric printer. A qualitative viability assay performed after the printing process showed high viability for both cell populations, proving the safety of the EmVP process. Overall, by employing a volumetric bioprinting system comprising multi‐color optics, was possible to fabricate highly featured multi‐material and multi‐cellular geometries, enhancing the accessibility of volumetric printing for advanced tissue models.

## 3 Conclusions

The unique approach of VBP, which combines the principles of tomographic manufacturing with the use of photoresponsive bioresins, offering ultra‐fast speed and precision in the fabrication of complex, cell‐laden living constructs, has provided new possibilities for large‐scale tissue engineering and regenerative medicine. The development of suitable bioresins, with defined and reproducible properties, is however a critical aspect that requires further exploration. The interplay of GelMA molecular weight, a vastly understudied parameter in bioprinting and light‐based bioprinting, DoM, temperature, and polymer concentration have a pivotal effect on the material printability and on the final mechanical properties. The precise tuning of photocross–linking kinetics is paramount to ensure accurate volumetric prints and avoid unwanted light dose accumulation, which could lead to over‐cross–linking and printing artifacts. Furthermore, the possibility of multi‐material printing and the production of complex tissues comprising multiple cell populations are significant hurdles that can be overcome to broaden the applicability of VBP. Further expanding the array of approaches that can volumetrically print complex biological structures as multi‐ or composite materials (i.e., embedding melt electrowetting‐produced opaque polymeric meshes), Embedded extrusion Volumetric Printing (EmVP) was recently developed. EmVP, which combines suspended bath bioprinting with VBP, has demonstrated the potential for positioning multiple cell types within a bioresin vat. However, the resolution of this technique is currently limited by the size of the microgels used. The use of gelatin as a sacrificial, thermoreversible viscosity enhancer offers a potential solution to this problem. However, the temperature‐dependent shear‐thinning behavior of commercially available gelatins restricts their use. In this study, the engineering of gelatin macromers was demonstrated as a promising strategy to overcome these limitations, thereby creating a library of fit‐for‐purpose materials for VBP, suspended bath printing, and EmVP. By varying the molecular weight and degree of modification of the gelatins, it is possible to produce bioresins with a wide range of characteristics and printability. The discovery of a subset of gelatins and GelMA that exhibit stable shear‐yielding behavior over extended periods represents a significant advancement, uniquely allowing them to act both as VBP‐resin, and suspension bath for embedded printing, without the need for a granular consistency (therefore improving resolution) or any complex additive or viscosity enhancer, by ultimately achieving tissue‐specific mechanical properties by adjusting the degree of modification. Moreover, our study offers a thorough characterization of the effect of multiple hydrogel parameters on printability with volumetric printing and embedded extrusion printing, offering a reference and blueprint for other researchers interested in producing resins and inks for these advanced fabrication technologies. This selection of materials is compatible for utilization in a multi‐color VBP printer to efficiently align different models to produce complex multi‐material and multi‐cellular geometries. In future developments, heterocellular and anisotropic tissue models that can be volumetrically produced in a high‐throughput fashion could have far‐reaching implications for biomedical and pharmaceutical research, paving the way for the next generation of in vitro tissue models.

## 4 Experimental Section

### GelMA Formulations

Porcine type A gelatins, used as non‐modified and modified formulations, were provided by Rousselot (Belgium). The name coding XpY refers as: X = MW, p = porcine, and Y = DoM.

### Photo‐Rheology for the Elucidation of the Tunability of GelMA Hydrogel Strengths

The GelMA types (160p80; 160p60; 160p40 & 90p80; 90p60, and 90p40), were dissolved in 22 mL amber vials at concentrations of 5, 10, 15, and 20% w/v in a PBS‐LAP stock‐solution, with LAP being lithium phenyl‐2,4,6‐trimethylbenzoylphosphinate, at a concentration of 1 mg mL^−1^ in a 1xPBS solution (0.01 m phosphate buffer, 0.0027 m potassium chloride and 0.137 m sodium chloride, pH 7.4, at 25 °C; Sigma, Belgium). The GelMA resins were placed in an oven (45 °C) for 1.5 h to improve solubility and they were subsequently vortexed. Next, the GelMA resins were placed in a vacuum chamber and a vacuum (50 mbar) was applied for 3 min until the foam collapsed. The samples were stored at 4 °C, in the dark.

The photo‐cross–linking kinetics of the GelMA resins were studied at 20 °C using an Anton Paar MCR 302e modular compact rheometer (Anton Paar, Belgium) that was equipped with an Omnicure S1500 light source, the wavelength of 365 nm could be selected by using a filter. Only for the photo‐rheology experiment reported in Figure 2, the test was conducted at 4, 21, and 37 °C to elucidate the effect of temperature on the cross–linking kinetics. The intensity was set to 10 mW cm^−2^ as measured at the quartz base‐plate using a DYMAX Accu‐Cal 50‐LED radiometer. The samples were in situ irradiated from the bottom through the quartz plate. A parallel plate setup (Ø 25 mm, gap 0.300 mm) was used. Frequency and strain sweeps were previously recorded and a shear frequency of 1 Hz and an amplitude (strain) of 1% were selected as they were within the linear viscoelastic range of the GelMA. The storage and loss moduli (*G*’/*G*’’) were monitored over time. The UV light was switched “on” at the 2 min mark and UV irradiation continued for 10 min. After UV irradiation the measurement was continued for an additional 5 min. The GelMA samples were stored in an oven at 45 °C during the rheology measurements.

### Dynamic Mechanical Analysis for the Elucidation of the Tunability of GelMA Hydrogel Strengths

The GelMA resins (160p80; 160p60; 160p40 & 90p80; 90p60, and 90p40), were dissolved to obtain solutions with a concentration of 5% w/v in a PBS‐LAP stock solution, consisting of 1x PBS (0.01 m phosphate buffer, 0.0027 m potassium chloride and 0.137 m sodium chloride, pH 7.4, at 25 °C; Sigma, Belgium), supplemented with 1 mg mL^−1^ lithium phenyl‐2,4,6‐trimethylbenzoylphosphinate (LAP). The GelMA resins were heated to 45 °C for one hour before cross–linking. The GelMA disks (Ø 6 mm, thickness 2 mm) were photo‐Cross–linked for 5 min, using an in‐house build curing chamber with a 365 nm 1 mW cm^−2^ light source, which had an intensity output of <1 mW cm^−2^, as measured at the array‐plate using a DYMAX Accu‐Cal 50‐LED radiometer. The Cross–linked GelMA disks were submerged in 1xPBS in the incubator (37 °C; 5% CO2) overnight. The mechanical properties of the GelMA hydrogels were tested with the Dynamic Mechanical Analyzer (DMA Q800, TA Instruments), and all tests were performed at room temperature. A quasi‐static compression (uniaxial, unconfined) was performed, consisting in a load phase at 0.1 N min^−1^ up to 0.3 N. The data was exported to MS Excel and the stress‐strain curves were used to calculate the compressive modulus (slope of the load curve in the linear range). To assess the viscoelastic properties, a strain recovery measurement was performed at a constant 20% strain for 2 min and then left for recovery for 1 min, with a preload force of 0.0010 N. The stress relaxation % was calculated as being the ratio between the recovered stress and the maximal stress under constant strain (value after 2 mins/highest value * 100).

### Rheology on the GelMA‐based support bath for embedded printing

The GelMA (160p60 and 90p60) and gelatin resins (160 and 90 kDa), were dissolved at concentrations of 5% w/v in a PBS‐LAP stock‐solution (1x PBS, 0.1 mg mL^−1^ LAP). Adjusted Bloom test: Gel casting: 30 mL of each GelMA or gelatin was added to a large petri dish (Ø 150 mm), obtaining a round surface with a thickness ≈1.7 mm of each product. The dish was then left at room temperature to physically gel for 16 h (overnight). After 16 h, small round sections were cut and transferred to the stainless‐steel base‐plate of the rheometer, a plate‐plate setup was used with a 25 mm plate spindle. A 1.65 mm gap was used. Paraffin oil and an evaporation cap were applied to prevent sample drying. A thixotropy test was applied to follow up on the storage and loss moduli (*G*’/*G*’’). The temperature was set to 21 °C, with a tolerance of 0.1 °C for 1 min to allow temperature equilibration. The *G*’ and *G*’’ were recorded during 16 h with a frequency of 1 Hz and shear strain of 1% (2 seconds per point). The shear rate ramp test was similarly performed. The shear stress and the viscosity were measured during a logarithmic shear rate ramp from 0.01 to 1000 s^−1^ (2 seconds per point). The shear stress ramp test was similarly performed, now using the shear strain during a logarithmic shear stress ramp from 0.01 to 1000 Pa (2 s per point). The stress recovery of the GelMA or gelatin was performed using a thixotropy test: similarly, the temperature to 21 °C (tolerance 0.1 °C for 1 min to allow temperature equilibration) and the *G*’ and *G*’’ were measured for 2 min with a frequency of 1 Hz and shear strain of 1% (2 s per point). Then a high shear rate was applied (500 s^−1^) to disrupt the gel for 2 min (2 seconds per point). After, the *G*’ and *G*’’ were again measured for 10 min, with a frequency of 1 Hz and shear strain of 1% (2 s per point).

### Soluble Fraction %

To assess sol‐fraction of the GelMA hydrogel formulation, cylindrical samples (7 mm diameter, 10 mm height, *n =* 3) were volumetrically printed at different temperatures (e.g., 4 °C and 21 °C) and were immediately lyophilized overnight after retrieval from the printing vial. The next day, the dry samples were weighed, and the dry mass (mass_dry,t0_) was measured. The samples were stored in deionized water to ensure rehydration of the dry gels and removal of the uncross–linked polymer fraction, and kept in an incubator at 37 °C overnight. Subsequently, samples were lyophilized for a second time overnight, and the mass of the dry samples was measured as mass_dry,t1_. The sol‐fraction formula of the hydrogel formulations for analysis of the cross‐linking properties of the GelMA formulations was calculated with the following formula:

(1) $$Sol-fraction\;\;\left[\%\right]=\frac{\mathrm{mas}s_{\mathrm{dry},t0}-\;\mathrm{mas}s_{\mathrm{dry},t1}}{\mathrm{mas}s_{\mathrm{dry},t0}}\times 100$$

### Porcine Pancreas Mechanical Characterization

A frozen porcine pancreas (−18 °C) was defrosted at 4 °C over 48h. The pancreas was used undissected and uncut and subjected to compression testing using a TA.XT plus texture analyzer (Stable Micro Systems Ltd, UK) at room temperature. A 13 mm diameter plunger was used. A trigger force of 0.005N was applied with an indentation depth of 5 mm at a speed of 0.5mm s^−1^. After reaching the designated depth the force was recorded for an additional 20 s, thereby providing information about the elasticity of the pancreatic tissue. 49 measurements were performed, covering all the areas of the pancreas in an evenly distributed fashion (a grid was used). To determine the compressive modulus, the recorded forces need to be divided over the area at which the force was recorded/applied. The diameter of the plunger is known (i.e., Ø 13 mm) hence strength could be calculated.

### Embedded Extrusion Printing

3D (bio)printing experiments were performed with a R‐GEN 100 (RegenHu, Switzerland), using a pneumatic‐driven extrusion printhead. For printing evaluation, 3 mL syringes were loaded with 1 mL of Cy‐3.5 stained GG/PEGDA bioink (already characterized in a recent work from our lab),^[^ ^9^ ^]^ two different needles (25G and 27G), and four different printing speeds (0.25, 0.5, 0.75, and 1 mm s^−1^) were tested to characterize the filaments' width. Images (*n =* 5) of S‐shape embedded filaments were taken with a confocal microscope (SPX8, Leica Microsystems, The Netherlands) and analyzed with ImageJ.

### Volumetric Printing

A commercial Tomolite v2.0 (Readily3D SA, Switzerland) volumetric bioprinter was used to fabricate the GelMA‐based constructs. The GelMA resins (160p80; 160p60; 160p40 & 90p80; 90p60, and 90p40), were dissolved at concentrations of 5% w/v in a PBS‐LAP stock‐solution (1x PBS, 0.1 mg mL^−1^ LAP) The GelMA formulations (cell‐free or embedding cells) were poured at 37 °C in Ø10 mm cylindrical glass vials and kept cool at 4 °C to ensure thermal gelation. User‐designed CAD files were loaded and processed using the Readily3D Apparite software. The average light intensity across all prints was set at 9.9 mW cm^−2^. After printing, the thermally gelated bioresin was washed with pre‐warmed PBS at 37 °C to remove the uncured GelMA from the printed structure.

### Multimaterial Sequential Volumetric Printing

A first print is performed as described above. The unpolymerized resin is washed out, and the vial containing the structure printed with the first material is then filled with the second material and the the volumetric printing process is repeated. As long as the optical properties of both materials are compatible with the printing process, a second print (overprinting) can be performed, and upon removal of the second, unreacted resin, a multi‐material object is formed. To perform this process correctly, ensuring the correct alignment of the first printed object leveraging a different wavelength prior to performing the second print was crucial. To prevent movement and displacement of the first print during the washing steps, it was resorted to printing an additional base at the bottom of the vial, to anchor the printed object in place. Then, the alignment of the projections of the model meant to be overprinted with the previously printed structure was performed, followed by the second volumetric printing step. The base can finally be removed at the end of the process, by simply cutting it with a razor blade.

### Embedded Extrusion Volumetric Printing (EmVP)

The extruded features and the model meant to be volumetrically overprinted were designed and saved separately as STL files. The GelMA resin 90p60 was dissolved at concentrations of 5% w/v in a PBS‐LAP stock‐solution, (1x PBS, 0.1 mg mL^−1^ LAP). First, a cylindrical vial was loaded with the 90p60 GelMA resin at 37 °C. The vial was left to thermally gelate at room temperature overnight, then placed in an R‐GEN 100 extrusion printer (RegenHu, Switzerland) and kept in place through a custom printed holder. 3 mL syringes were loaded with 1 mL of (bio)ink and the G‐codes of the extruded features were manually written. Subsequently, the vial was loaded into a custom‐made Polychromatic Tomolite volumetric printer (Readily3D SA, Switzerland). To align the extruded features with the model intended to be overprinted, a first series of light‐projections was delivered on the rotating vial with a 520 nm laser, orthogonal wavelength to the cross–linking. Therefore, 2D light‐projections of the structure meant to be volumetrically printed were delivered with a 405 nm laser, until the curing of the fluid support bath (90p60) was achieved. Finally, the vial was heated to 37 °C to dissolve the unpolymerized GelMA and the sample was retrieved and washed with prewarmed PBS. For multicellular extrusion printing, iβ‐cells (10 × 10^6^ mL^−1^) and hMSC (5 × 10^6^ mL^−1^) were pre‐stained (DiL and Did, respectively. Vybrant Multicolor Cell‐Labeling Kit, Thermo Fischer Scientific, The Netherlands) printed with a 25G needle (250 µm diameter).

### Volume Comparison for Printing Accuracy

To visualize and generate the 3D reconstructions of the printed models, GelMA formulations were stained with a fluorescent dye (Cy 3.5 Phosphoramidite), and light‐sheet microscopy was used to scan them. Once the 3D‐printed structures are manufactured, they are scanned using a customized image system available at our facilities. Different images are obtained once the 3D model is scanned and they are gathered to reconstruct the 3D model using ImageJ. After that, post‐processing imaging is carried out to remove outliers and unwanted parts. After that, a detailed quantitative analysis was carried out to calculate the differences between the real STL and the 3D‐printed model, using a point‐based registration of the meshes. For that, CloudCompare open‐source software was used. Both STL files were aligned and the distance between both meshes was computed using the cloud‐to‐cloud distance, which is the Hausdorff distance algorithm.

### Cells Isolation and 2D culture

iβ‐cells were obtained and cultured as previously described.^[^ ^24^ ^]^ Briefly, a 1.1E7 cell line‐derived cell clone deficient in glucose‐sensitive insulin secretion was transduced with Proinsulin‐NanoLuc‐derived lentiviral particles and selected in a culture medium containing 5 µg mL^−1^ blasticidin to create polyclonal INSvesc cells. Next, a monoclonal cell population was picked based on the best performance for depolarization‐triggered nanoLuc secretion. This cell line was co‐transfected with the SB100X expression vector pCMV‐T7‐SB100 (PhCMV‐SB100X‐pA) and the SB100X‐specific transposon pMMZ197 (ITR‐PhEF1α‐melanopsin‐pA‐PRPBSA‐ypet‐P2A‐PuroR‐pA‐ITR) to generate a polyclonal population of iβ‐cells that stably expressed melanopsin as well as Proinsulin‐NanoLuc cassettes. After selection for two weeks in a medium containing 5 µg mL^−1^ blasticidin and 1 µg mL^−1^ puromycin, the monoclonal iβ‐cells were sorted by means of FACS (Becton Dickinson LSRII Fortessa flow cytometer) and screened for blue‐light‐responsive nanoLuc secretion. iβ‐cell were cultured in RPMI 1640 Medium, GlutaMAX, HEPES (Gibco, Life technologies) supplemented with FBS (10% v/v) and 1% P/S and used at passage 3–4. cells were cultured in a 95% humidified incubator at 37 °C, 5% CO2.

Human bone marrow‐derived mesenchymal stromal cells (MSCs) were isolated from bone marrow aspirates of consenting patients, as previously described.^[^ ^9^ ^]^ Briefly, human bone marrow aspirates were obtained from the iliac crest of patients who were receiving spondylodesis or hip replacement surgery. Isolation and distribution were performed in accordance with protocols approved by the Biobank Research Ethics Committee (isolation 08‐001, distribution protocol 18–739, University Medical Center Utrecht). The protocols used were in line with the principles embodied in the Declaration of Helsinki. MSCs were expanded in α‐Modified Eagle Medium culture medium (α‐MEM, Gibco, Life Technologies) supplemented with 10% FBS, 1% P/S, 1% L‐ascorbic acid‐2‐phosphate (ASAP; Sigma–Aldrich, The Netherlands), and 1 ng ml^−1^ basic fibroblast growth factor (bFGF; R&D Systems) and used at passage 4–5.

### Statistical Analysis

Results were reported as mean ± standard deviation (S.D.). Statistical analysis was performed using GraphPad Prism 8.0.2 (GraphPad Software, USA). Comparisons between multiple (>2) experimental groups were assessed via one or two‐way ANOVAs, followed by post hoc Bonferroni correction to test differences between groups. Student's t‐test was performed between 2 experimental groups for statistical analysis. An unpaired t‐test was used for parametric comparisons of data sets. Non‐parametric tests were used when normality could not be assumed. Differences were considered significant when *p* < 0.05. Significance is expressed on graphs as follows: * *p* ≤ 0.05, ** *p* ≤ 0.01, *** *p* ≤ 0.001, **** *p* ≤ 0.0001.

## Conflict of Interest

The authors J.P.Z., T.V.G. and J.O. are employed at Rousselot BV, which commercializes gelatins and GelMA. P.D. is an employee and stakeholder of Readily3D SA, a manufacturer of volumetric 3D printers. R.L. is the scientific advisor for Readily3D SA.

## Supporting information



Supporting Information

## Data Availability

The data that support the findings of this study are available from the corresponding author upon reasonable request.
