# Supplementary material for: Multi‐material Volumetric Bioprinting and Plug‐and‐play Suspension Bath Biofabrication via Bioresin Molecular Weight Tuning and via Multiwavelength Alignment Optics
Source: Adv Mater. 2025 Feb 26;37(13):2409355. doi: 10.1002/adma.202409355 (PMC11962684; doi:10.1002/adma.202409355)
Supplement: Supplementary file 1 — Supporting Information [file ADMA-37-2409355-s001.docx]

**Multi-material volumetric bioprinting and plug-and-play suspension bath biofabrication via bioresin molecular weight tuning and via multiwavelength alignment optics**

Davide Ribezzi^1^, Jan-Philip Zegwaart^1,2^, *Thomas Van Gansbeke*^2^, Aitor Tejo-Otero^3,4^, Sammy Florczak^1^, Joska Aerts^1^, Paul Delrot^5^, Andreas Hierholzer^6^, Martin Fussenegger^6,7^ Jos Malda^1,3^, Jos Olijve^2^, Riccardo Levato^3,1^*

^1^ Department of Orthopaedics, University Medical Center Utrecht, Utrecht University, 3584 CX, Utrecht, The Netherlands.

^2^ Rousselot, Port Arthurlaan 173, 9000 Gent, Belgium.

^3^ Department of Clinical Sciences, Faculty of Veterinary Medicine, Utrecht University, 3584 CT, Utrecht, the Netherlands.

^4^ BIOMAT Research Group, University of the Basque Country (UPV/EHU), Escuela de Ingeniería de Gipuzkoa, Plaza de Europa 1, 20018 Donostia-San Sebastián, Spain.

*^5^Readily3D SA, EPFL Innovation Park, Building A, Lausanne, CH-1015 Switzerland*

^6^ Department of Biosystems Science and Engineering, ETH Zurich, Mattenstrasse 26, Basel CH-4058, Switzerland.

^7^ Faculty of Science, University of Basel, Mattenstrasse 26, CH-4058 Basel, Switzerland.

*Correspondence to: [r.levato@uu.nl](mailto:r.levato@uu.nl)

**SUPPLEMENTARY MATERIAL AND FIGURES**

**1. MW and DoM interplay within GelMAs formulations results in a wide range of mechanical properties**


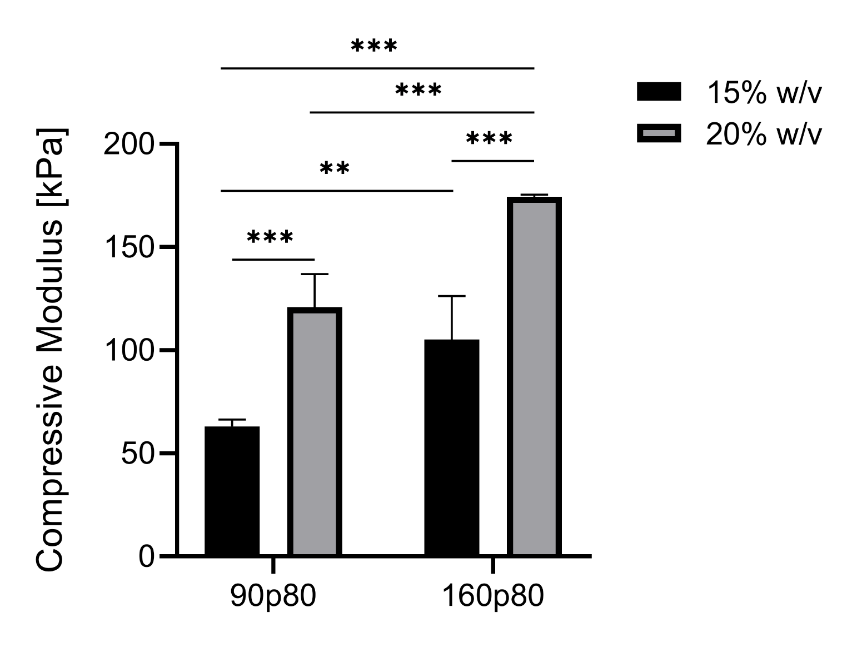


**Supplementary Figure S1: Compression modulus of formulations with densest hydrogel networks.** Compressive modulus of GelMA 90p80 and 160p80 at 15% and 20% w/v concentrations. Hydrogels crosslinked upon exposure to the same light dose provided in the rheometer are formed correctly, are highly stiff and do not soften during the photo exposure.

|  |  | | | | | | | | | | | |
| --- | --- | --- | --- | --- | --- | --- | --- | --- | --- | --- | --- | --- |
| wt% | **90p40** | **SD** | **90p60** | **SD** | **90p80** | **SD** | **160p40** | **SD** | **160p60** | **SD** | **160p80** | **SD** |
| 5 | 701 | 62 | 1.353 | 89 | 2.560 | 210 | 3.819 | 430 | 4.483 | 233 | 14.628 | 353 |
| 10 | 9.543 | 580 | 17.674 | 1.328 | 31.838 | 142 | 23.339 | 1.019 | 28.065 | 673 | 81.022 | 1.152 |
| 15 | 30.033 | 1.575 | 52.633 | 1.066 | 80.930 | 10.154 | 51.856 | 3.117 | 63.069 | 845 | 170.320 | 1.279 |
| 20 | 60.299 | 955 | 99.183 | 5.662 | 98.245 | 72.625 | 80.418 | 926 | 97.275 | 1.745 | 232.563 | 31.740 |

**Table S1: Storage Modulus G' [Pa] mean values and standard deviations after 10 minutes of illumination (0.1%w/v LAP, 10mW/cm^2^, PBS 1x, 20°C)**

**2. Effect of light source intensity on crosslinking kinetics**


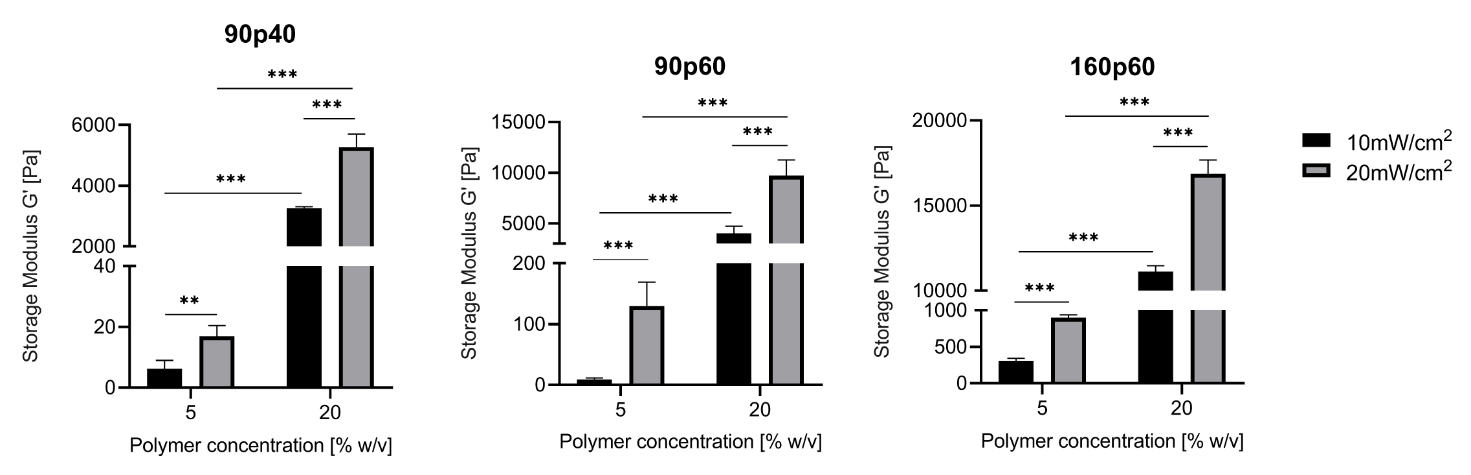


**Supplementary Figure S2: Effect of light source intensity on GelMAs crosslinking kinetics.** Storage modulus at the 2.5 minutes mark of GelMA 90p40, 90p60 and 160p60, at 5% and 20% w/v concentrations, crosslinked with light intensities of 10 and 20 mW/cm^2^. Higher irradiation intensity leads to a faster progression of the gelation, and therefore, in higher storage modulus values compared to gels exposed to lower light intensities, if the irradiation time is kept constant (in this case, 30 seconds after starting the light exposure, as this timing is comparable to the printing time in the volumetric printer for GelMAs).

**3. Influence of the photo-initiator concentration and salt content on photocrosslinking**

To correctly set a reproducible hydrogel crosslinking protocol that could be shared, a comparative evaluation of different approaches commonly used in the literature to calculate the LAP photo-initiator concentrations was performed. Three concentrations methods can be discerned from the literature, namely:

1. the stock-solution concentration method (i.e., a standard solution is used for all conditions, e.g., 1 mg/mL of LAP). This is also one of the most used approach in the literature. As a consequence of this method, the ratio between the PI concentration and that of the reactive groups will be different once the GelMA type and concentrations are varied;
2. the mass-ratio concentration method (i.e., the amount of LAP is proportional to the amount of GelMA in the resin, e.g., 1 mg of LAP per 1 g of GelMA);
3. the mol% concentration method (i.e., the amount of LAP is proportional to the methacryloyl content of the GelMA resin, e.g., 2 mol % of LAP relative to the methacryloyl content)

To exemplify what these different concentration methods entail, a numerical example is provided.

1. The stock-solution concentration method:

To create a 3 mL GelMA-resin at 5% w/v and 0.1% w/v LAP (1 mg/mL):

| GelMA-type [DoM] | Weight/Volume % | Total volume Resin [mL] | GelMA [mg] | LAP [mg] | Mol% LAP |
| --- | --- | --- | --- | --- | --- |
| 40 | 5 | 3 | 150 mg | 0.1 w/v % of 3 mL = 3 mg LAP | 48.6 |
| 60 |  |  |  |  | 32.4 |
| 80 |  |  |  |  | 24.3 |

If a 3 mL GelMA-resin at 10% w/v and 0.1% w/v LAP (1 mg/mL) is produced:

| GelMA-type [DoM] | Weight/Volume % | Total volume Resin [mL] | GelMA [mg] | LAP [mg] | Mol% LAP |
| --- | --- | --- | --- | --- | --- |
| 40 | 10 | 3 | 300 mg | 0.1 w/v % of 3 mL = 3 mg LAP | 24.3 |
| 60 |  |  |  |  | 16.2 |
| 80 |  |  |  |  | 12.1 |

The issue with this approach is that the mol% LAP changes both with degree of modification (DoM) and with w/v% of the GelMA-resin (to correct for w/v% you would need to use 0.2 w/v% LAP for the 10 w/v% GelMA). In terms of curing kinetics, the ability to compare resins with one another is lost.

1. The mass-ratio concentration method:

To create a 3 mL GelMA-resin at 5% w/v and 10% w/v using a mass-ratio of 1 to 1000 for LAP to GelMA the variability across w/v% disappears.

| GelMA-type [DoM] | Weight/Volume % | Total volume Resin [mL] | GelMA [mg] | LAP [mg] | Mol% LAP |
| --- | --- | --- | --- | --- | --- |
| 40 | 5 | 3 | 150 mg | 0.15 mg LAP (1:1000 ratio to GelMA mass) | 2.43 |
| 60 |  |  |  |  | 1.62 |
| 80 |  |  |  |  | 1.21 |

If a 3 mL GelMA-resin at 10% w/v and a 1:1000 ratio LAP:GelMA is applied, the following amounts and mol% are required:

| GelMA-type [DoM] | Weight/Volume % | Total volume Resin [mL] | GelMA [mg] | LAP [mg] | Mol% LAP |
| --- | --- | --- | --- | --- | --- |
| 40 | 10 | 3 | 300 mg | 0.30 mg LAP (1:1000 ratio to GelMA mass) | 2.43 |
| 60 |  |  |  |  | 1.62 |
| 80 |  |  |  |  | 1.21 |

The variability across w/v% of GelMA disappears. However, variability across DoM still exists.

1. The mol% concentration method:

To create a 3 mL GelMA-resin at 5% w/v and 10% w/v using the mol% concentration method allows for a comparison across w/v% of GelMA at their respective DoM. The amount of photo-initiator is correlated to the amount of methacryloyl-groups.

| GelMA-type [DoM] | Weight/Volume % | Total volume Resin [mL] | GelMA [mg] | LAP [mg] | Mol% LAP |
| --- | --- | --- | --- | --- | --- |
| 40 | 5 | 3 | 150 mg | 0.124 | 2 |
| 60 |  |  |  | 0.183 | 2 |
| 80 |  |  |  | 0.247 | 2 |

For a 3 mL GelMA-resin at 10% w/v the following LAP amounts are required:

| GelMA-type [DoM] | Weight/Volume % | Total volume Resin [mL] | GelMA [mg] | LAP [mg] | Mol% LAP |
| --- | --- | --- | --- | --- | --- |
| 40 | 10 | 3 | 300 mg | 0.247 | 2 |
| 60 |  |  |  | 0.371 | 2 |
| 80 |  |  |  | 0.494 | 2 |

The effect of using the stock-solution concentration method, the mass-ratio and the mol% *photo-initiator* (PI) concentration method in GelMA hydrogel production were studied using photo-rheology (Figure SI-1 and Figure SI-2).

In Supplementary Figure S1 the 90 kDa GelMAs are presented in the left panels, the 160 kDa GelMAs are presented in the right panels. The orange and red curves in Figure SI-1 show the effect of using the PI stock-solution approach. In all graphs photo-crosslinking is completed within the first 2 minutes of UV/vis irradiation (i.e., at an energy dose of 888 mJ). Interestingly, in the 90p40 GelMA condition at a concentration of 5 % w/v, no crosslinking is seen in the mass-to-mass condition (2.43 mol%) or the mol% condition (2 mol%), i.e., an PI amount of 48.6 mol% did incite crosslinking; these data suggest there is an lower limit in which crosslinking can be incited or not (Figure SI-1: panel A).

Furthermore, in all panels of Supplementary Figure S1 it is noted that the light and dark green curves (i.e., indicating the mass-to-mass concentration method) show a delay in curing onset, whereas the light blue and dark blue curves do not show this retarded effect. If this late onset effect was the result of lower PI amounts, than the blue curve in Figure SI-1: panel B should have shown a later curing onset point than the green curve, as the LAP amounts are greater in that variant for the mass-to-mass (2.43 mol%) approach than the mol% approach (2 mol%), however this is not the case. Moreover, the onset in panel C is much later for the 90p60 mass-to-mass approach (1.62 mol%) than for the 90p80 mass-to-mass approach (1.21 mol%), even though the PI amounts are lower in the latter case. Currently it is not clear why this “late curing onset” exists, especially as it is only seen in the mass-to-mass conditions.


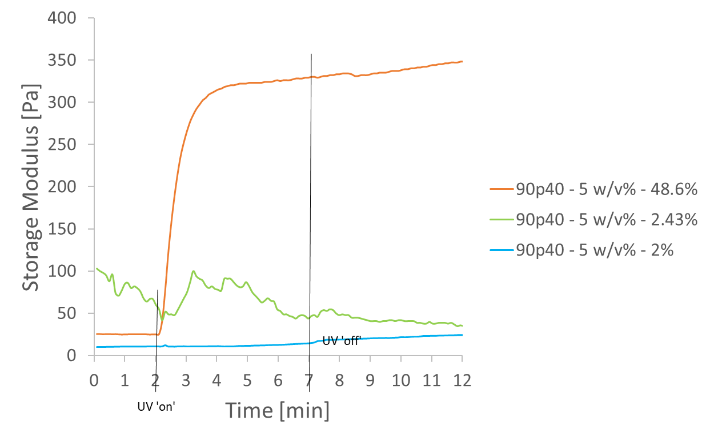

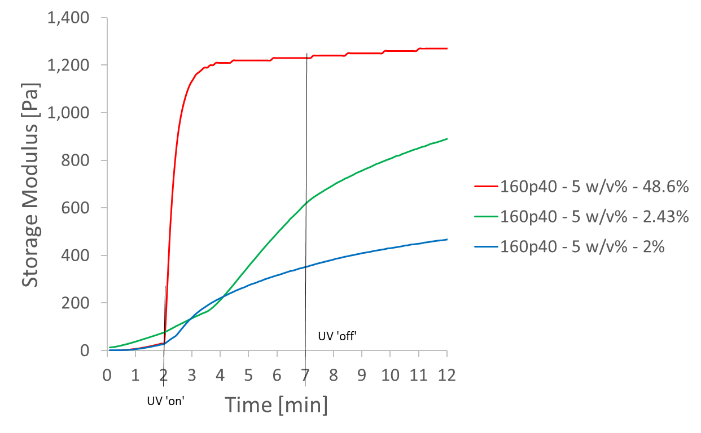


B

A


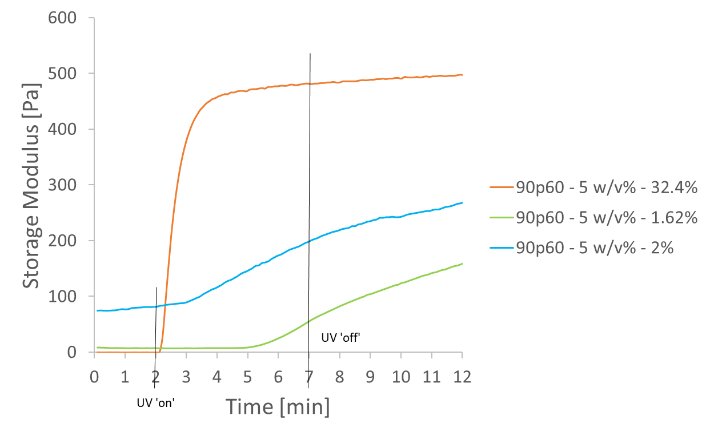

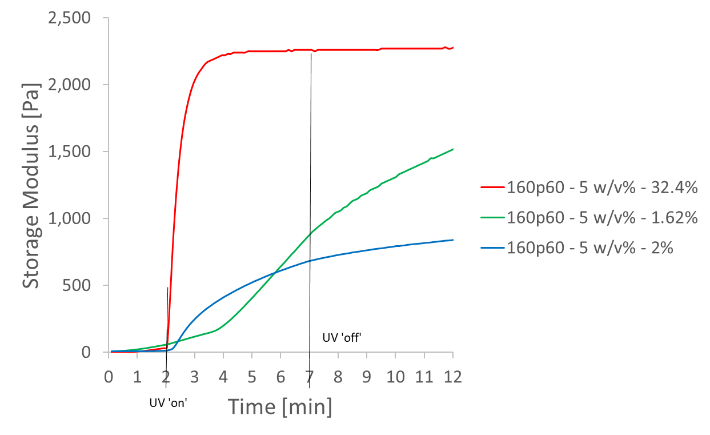


D

C


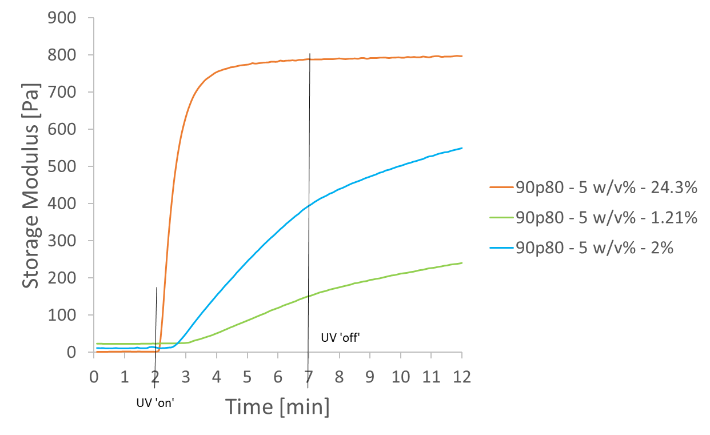

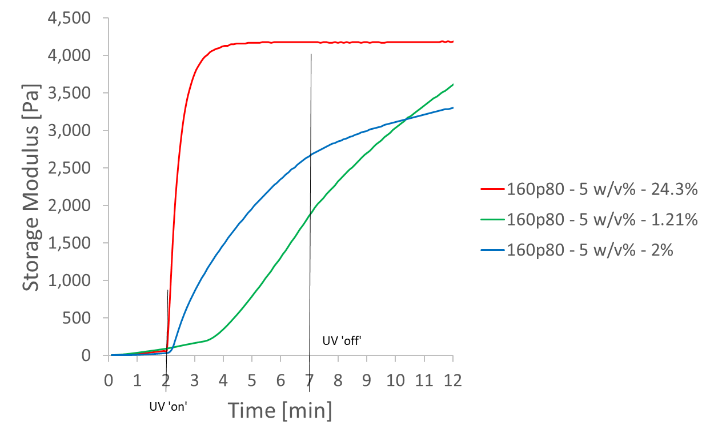


F

E

**Supplementary Figure S3:** Comparison of storage moduli between using the stock-solution or the mass-to-mass or the mol% concentration methods. The subsequential photo-initiator mol% are given in the graphs. A: GelMA 90p40 at 5 % w/v; B: GelMA 160p40 at 5 % w/v; C: GelMA 90p60 at 5 % w/v; D: GelMA 160p60 at 5 % w/v; E: GelMA 90p80 at 5 % w/v; F: GelMA 160p80 at 5 % w/v.

Supplementary Figure S2 shows the rheology curves of the GelMA conditions at 10 % w/v concentrations. As with Supplementary Figure S1, the stock-solution conditions show fast curing kinetics, with complete crosslinking within the first 2 minutes of UV/vis irradiation. Remarkably, panels B, C, D and F of Supplementary Figure S2, show higher obtained storage moduli for the mass-to-mass PI concentration approach, i.e., the curing kinetics are slower than the stock-solution approach, but the curing is sustained for much longer, resulting in greater hydrogel strength. This effect of slower curing kinetics, but more sustained curing, is most pronounced in the 160p80 condition (Supplementary Figure S2F).


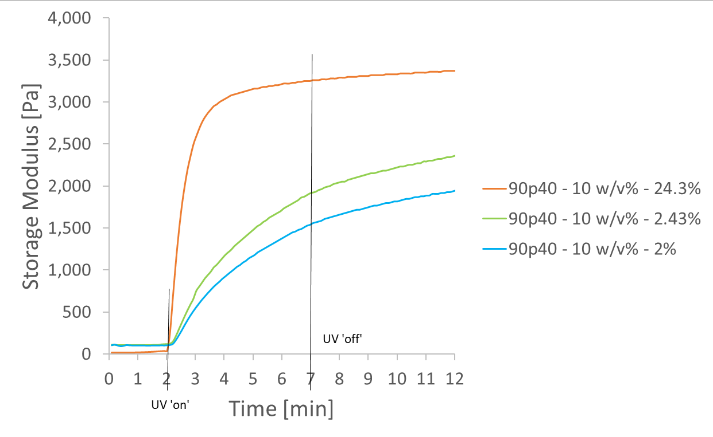

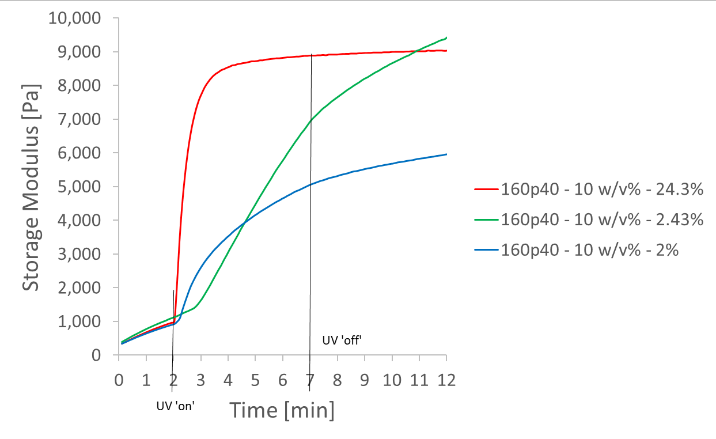


B

A


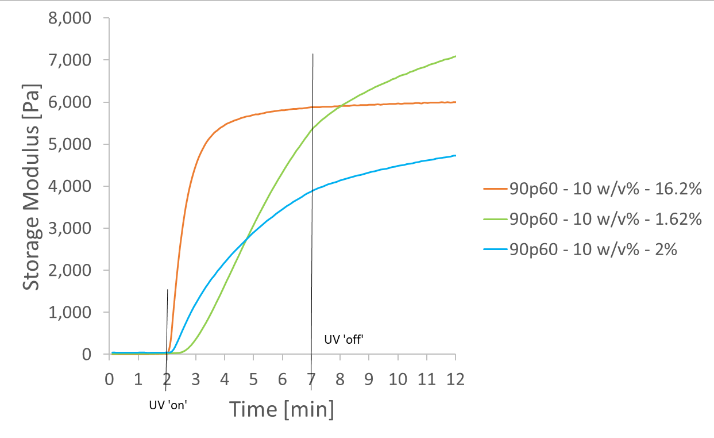

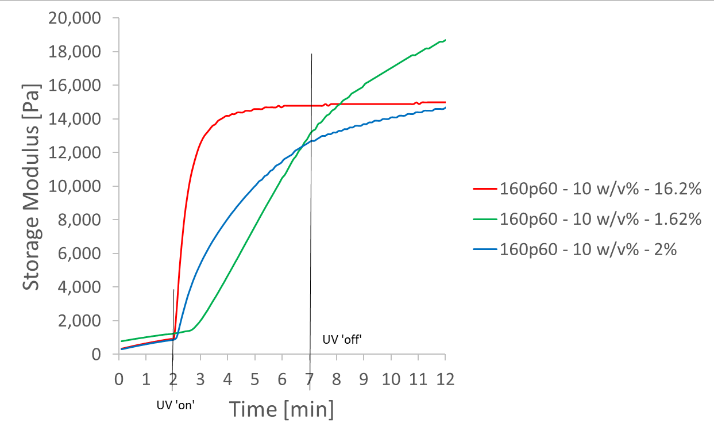


D

C


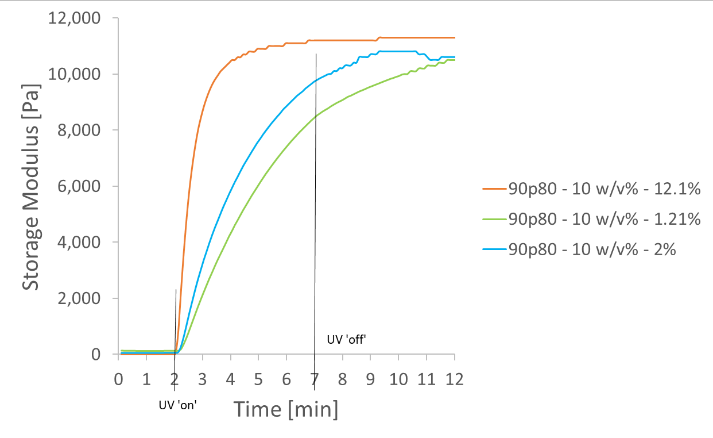

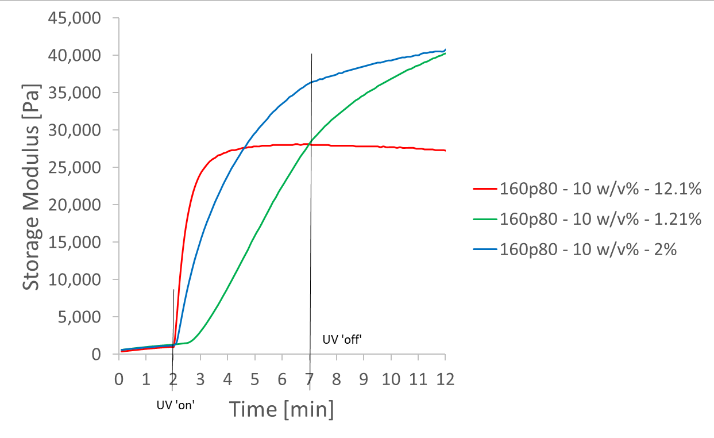


F

E

**Supplementary Figure S4:** Comparison of storage moduli between using the stock-solution or the mass-to-mass or the mol% concentration methods. The subsequential photo-initiator mol% are given in the graphs. A: GelMA 90p40 at 10 % w/v; B: GelMA 160p40 at 10 % w/v; C: GelMA 90p60 at 10 % w/v; D: GelMA 160p60 at 10 % w/v; E: GelMA 90p80 at 10 % w/v; F: GelMA 160p80 at 10 % w/v.

The importance of describing the applied photo-initiator concentration method in developing GelMA-based bioresins cannot be overestimated with respect to curing kinetics, hydrogel strength, but also debatably, on curing on-set. Three concentration methods were discussed, namely the stock-solution method, the mass-to-mass concentration method and the mol% concentration method. The stock-solution method results in simultaneous variations in w/v% and photo-initiator concentrations, without regard for both the w/v% concentrations of the resins nor the DoM. The mass-to-mass concentration method introduces a fixed ratio between w/v% and photo-initiator (typically 1000:1), but does not take the DoM into account. The mol% concentration method does consider both w/v% and the DoM. The data suggest, there is a minimal photo-initiator amount that is needed to start the crosslinking process, as shown in the 90p40 at 5 % w/v conditions. The data also confirms that with increasing concentration of LAP photo-initiator the curing kinetics will increase, while simultaneously it is shown that a continued increase in LAP photo-initiator concentration leads to a plateau in curing kinetics and even cause a decrease in mechanical strength, i.e., storage modulus of the GelMA hydrogels. Furthermore, considering the impact of this selection when formulating a resin for volumetric bioprinting, each method will lead to a resin with a different ratio between the amount of LAP and the amount of reactive methacryloly groups. This in turn will also affect the optimal light dose for printing, as the crosslinking velocity will also vary as a function of the LAP-to-methacryloyl groups ratio. The stock-solution concentration method is, however, the most adopted in the field when describing bionks and bioresins formulations for light-based bioprinting. Therefore, our experiments reported in Figures1-5 were conducted with this method, as we find this data to be most relevant for most users. It should be noted that, when selecting the stock-solution method, since the LAP content is always fixed (at 0.1 mg mL^-1^ in our case) and not proportional to the actual content of reactive MA groups, the light dose needed for printing will need to be adjusted for each formulation, something that could potentially be avoided using a different preparation method.

Finally, in Supplementary Figure S3, an example is given using various concentrations of phosphate-buffered saline (PBS) as solution medium for GelMA polymers. PBS is a typical buffer solution used in biological research. It is a water-based salt solution containing disodium hydrogen phosphate, sodium chloride and, in some formulations, potassium chloride and potassium dihydrogen phosphate. Typically, PBS buffers are denoted as 1x or 10x PBS, indicating the concentration levels of the various salts that are present. Supplementary Figure S3, shows that with increasing concentrations of PBS the hydrogel strength decreases, as represented by the storage modulus. By varying the PBS concentration from 0.01x to 1x a decrease of about 60% (from ~13 kPa to ~5 kPa) is shown for the 160p60 GelMA at 5% w/v. The 10x PBS interfered with the photo-curing considerably, the hydrogel produced was not stable, as is clearly visible from the downward trailing of the curve (lightest hue blue curve).


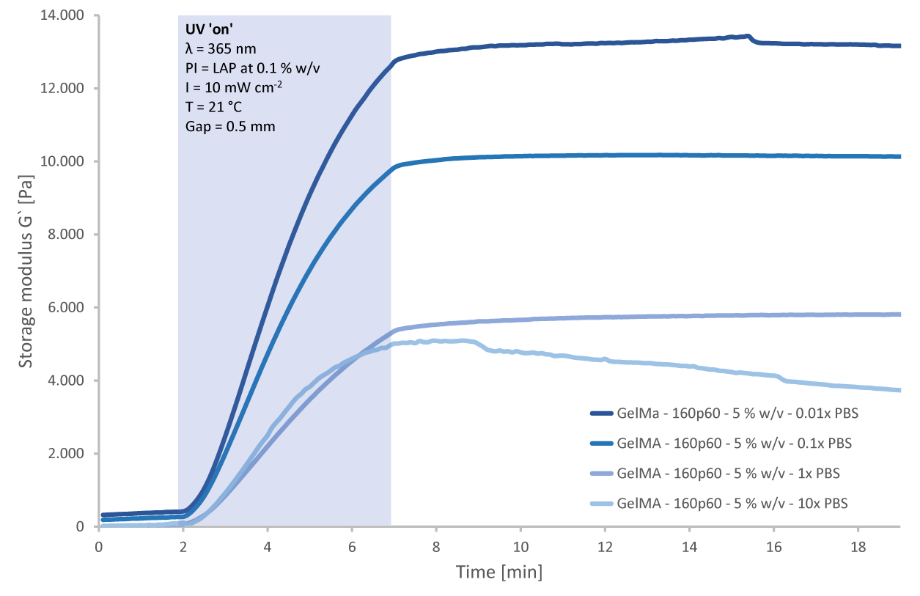


**Supplementary Figure S5:** Effect of salt concentration on gelMA mechanical properties. With an increase of concentrations of PBS, the hydrogel strength decreases, as represented by the storage modulus.

**4. Effect of printing temperature on final crosslinking density**


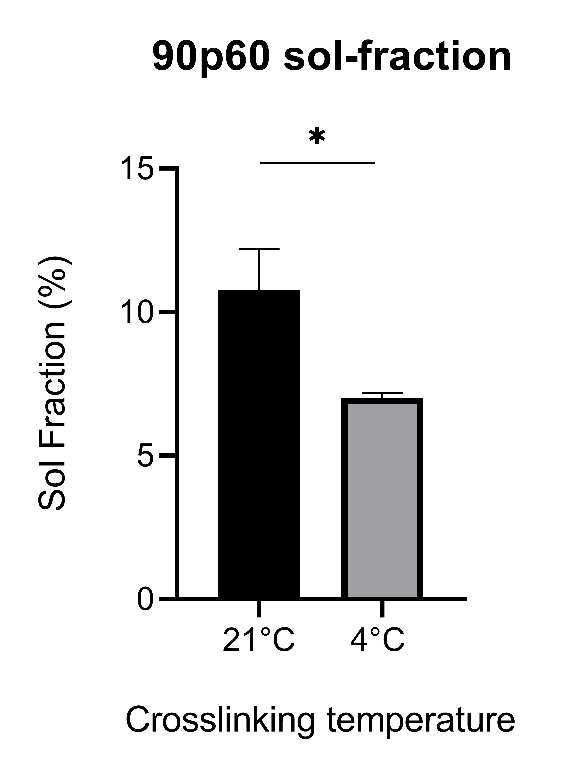


**Supplementary Figure S6: GelMA 90p60 sol-fraction % at different crosslinking temperatures.**

A significantly lower sol-fraction % is measured on samples crosslinked at 4°C (7%), compared to crosslinked samples at 21°C (10.8%). This could be attributed to a higher (faster) conversion of MA when crosslinking at lower temperatures.

**5. Cell encapsulation and bioprinting**

­­
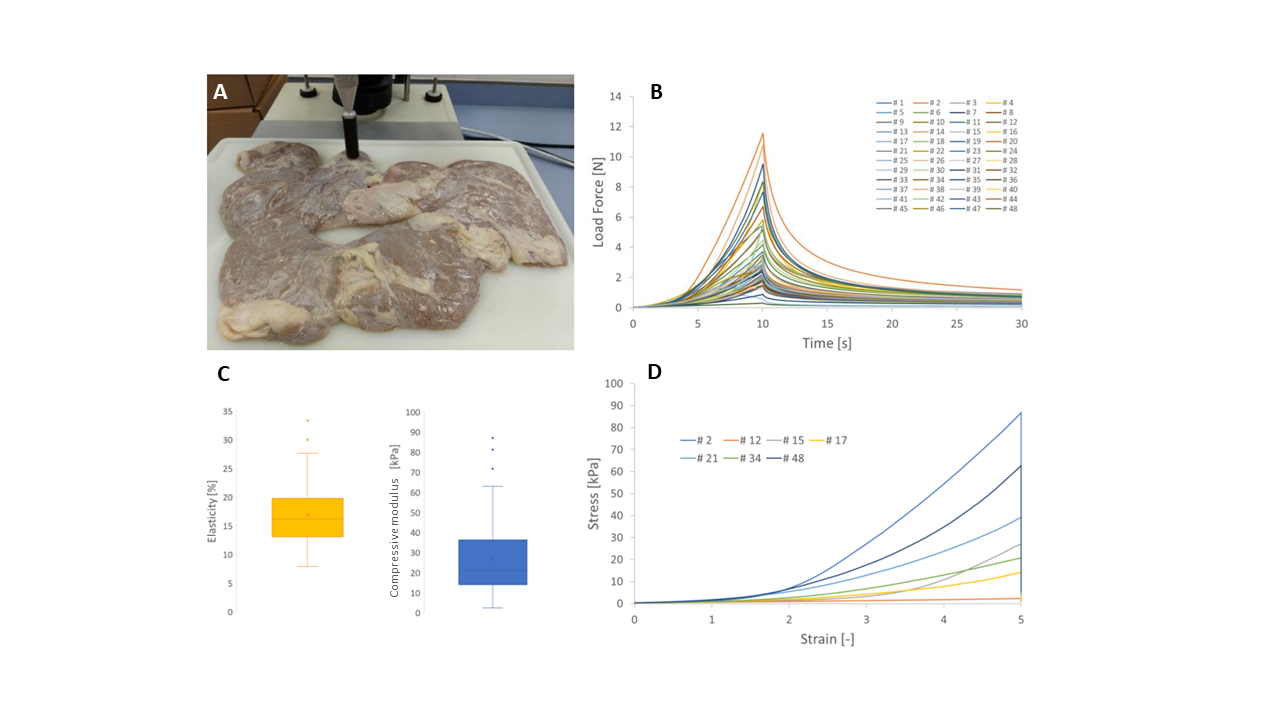


**Supplementary Figure S7: Porcine pancreatic tissue mechanical characterization.** A) Porcine pancreas, ready for compression test using an indentation system. B) Recordings of Force as a function of time. The tissue was compressed over a fixed distance of 5 mm after the trigger force of 0.05 N had been exceeded. C) Yellow panel: elasticity of pancreatic tissue. Blue panel: compressive modulus of pancreatic tissue. D) Stress-strain curves of the maximum (#2), minimum (#12), transitions Q1-4 (#17, #21 and #48), average (#15) and median (#34) compression measurements.

**
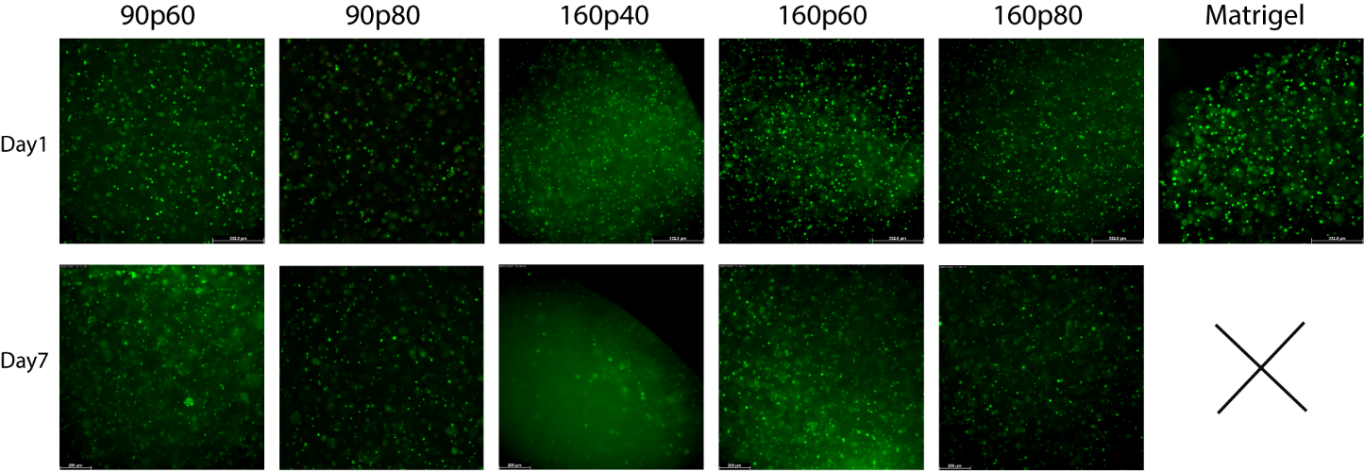
**

**Supplementary Figure S8:** iβ-cells viability screening after embbeding in different gelMA formulations and Matrigel. Live/Dead images showing good cell viability. Geltrex could not be imaged on day 7 as it was degraded. First clusters start to appear after 7 days in GelMA 90p60 Scale bar: 300µm

­


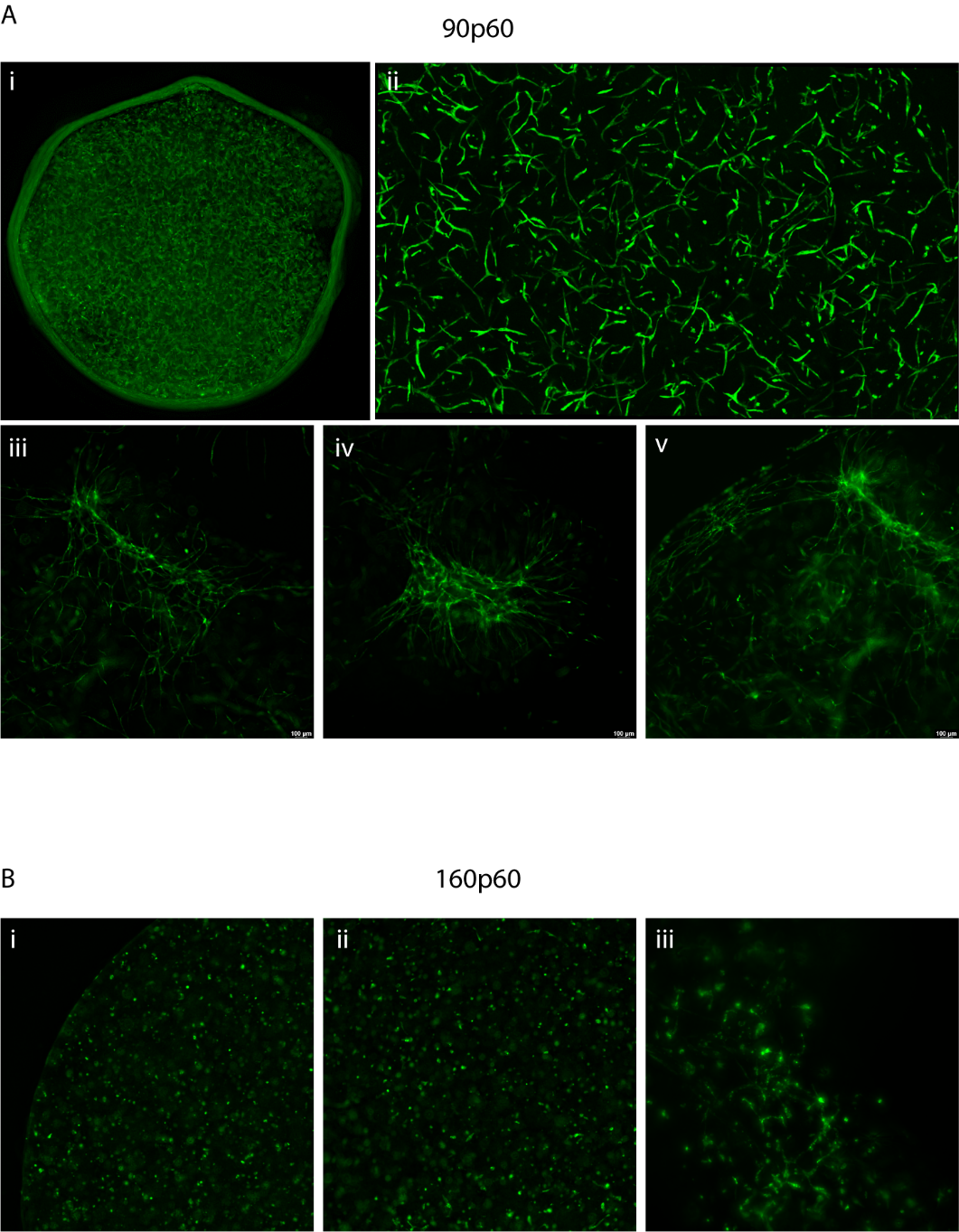

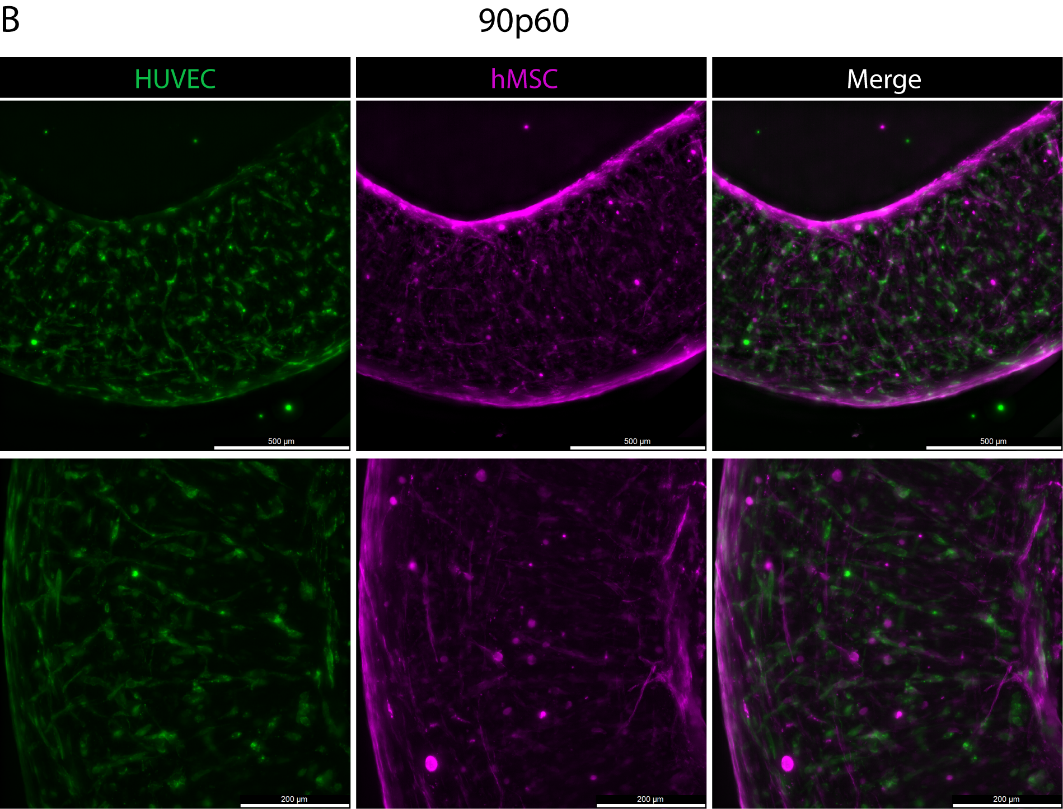

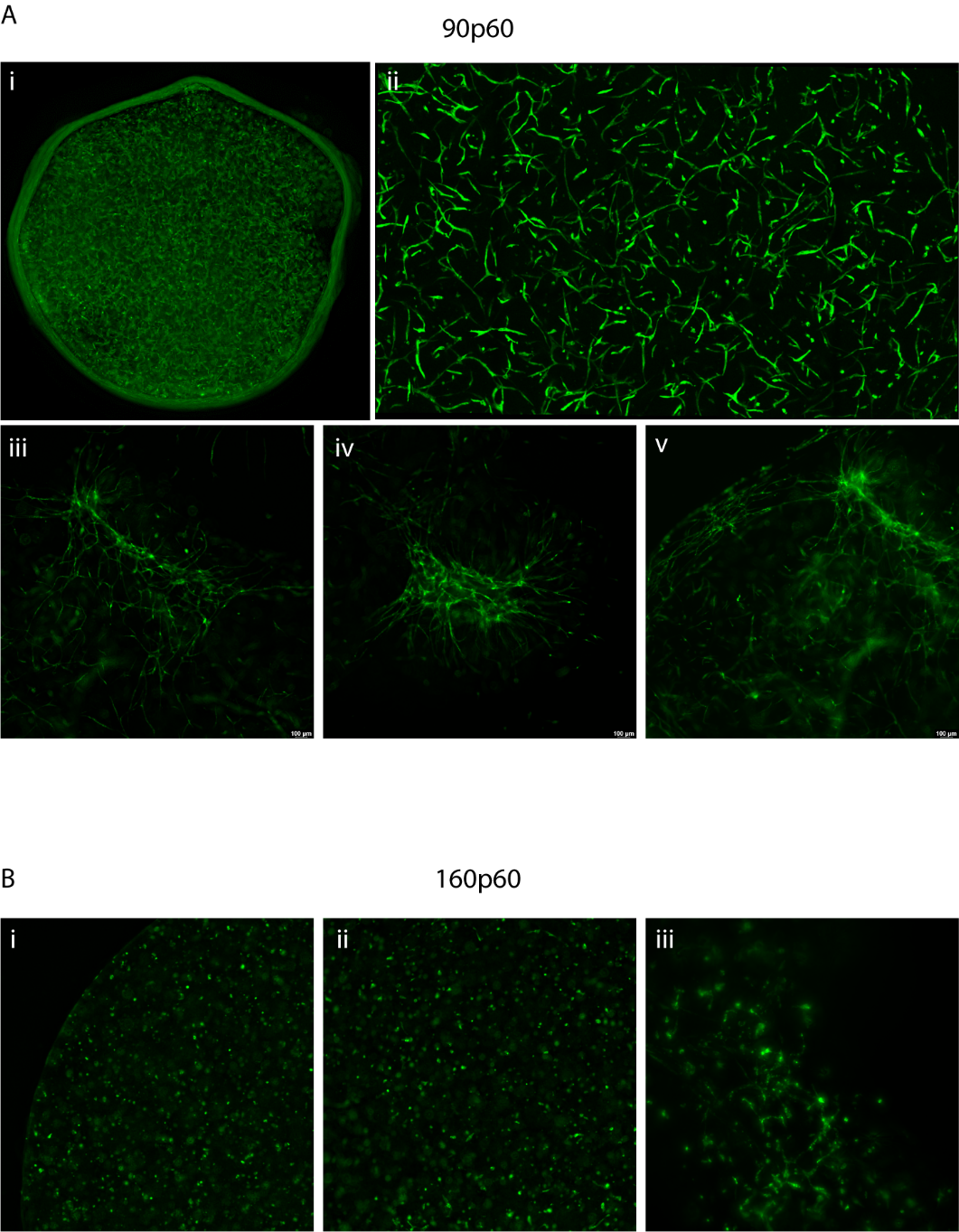


C

HUVEC

HUVEC

**Supplementary Figure S9:** Co-culture of HUVEC and hMSC cells in 90p60 and 160p60 (5%w/v polymer concentration, 0.1% w/v LAP concentration) at day 7. The first capillaries start to appear at day 7 only in GelMA 90p60. A) GPF-labelled HUVEC in GelMA 90p60. B) GPF-labelled HUVEC and α-Smooth Muscle Actine (α-SMA) stained hMSC in GelMA 90p60. C) GPF-labelled HUVEC in GelMA 160p60. Ai) Disc diameter: 6mm. Zoomed pictures: Aii; Bi,ii : 5x magnification. Aiii,iv,v; Biii : 10x magnification.

**6. Printing of complex and multimaterial structures**


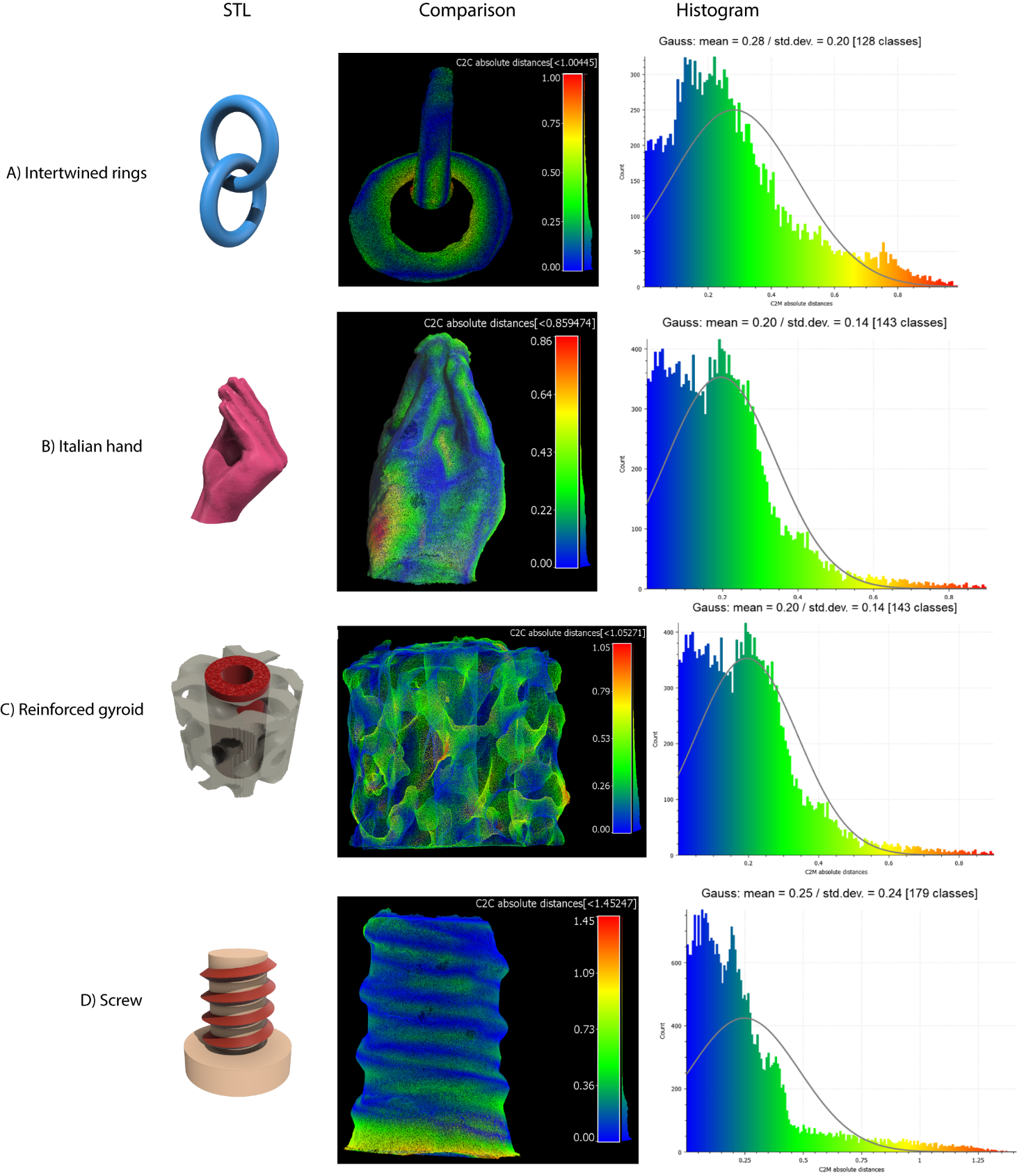


**Supplementary Figure S10: Quantitative printing accuracy.** STL models, 3D maps providing the difference between the original STL file and histograms depicting the size variations in mm of the volumetrically printed models: A) intertwined rings, B) Italian hand, C) Reinforced gyroid and D) Screw.


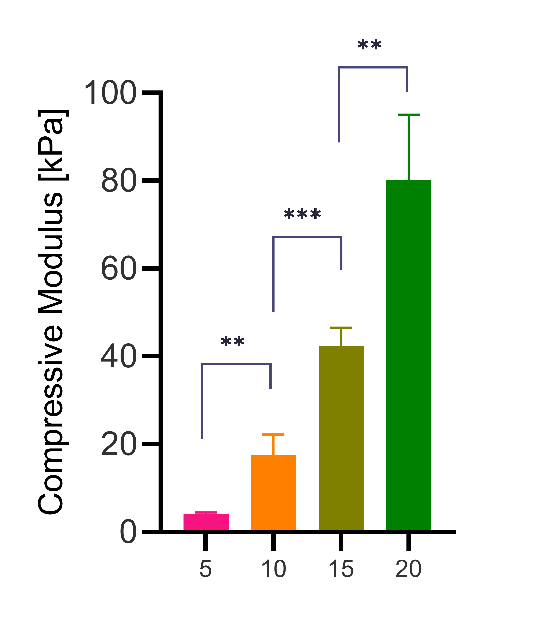


**Supplementary Figure S11:** Compressive modulus of gelMA 160p60 at different concentrations. In combination with lower molecular weight gelMA based formulations, 160p60 can act as mechanical support in the multimaterial model.

**7. Formation of optimal suspension baths for embedded printing using gelatins at different MW**


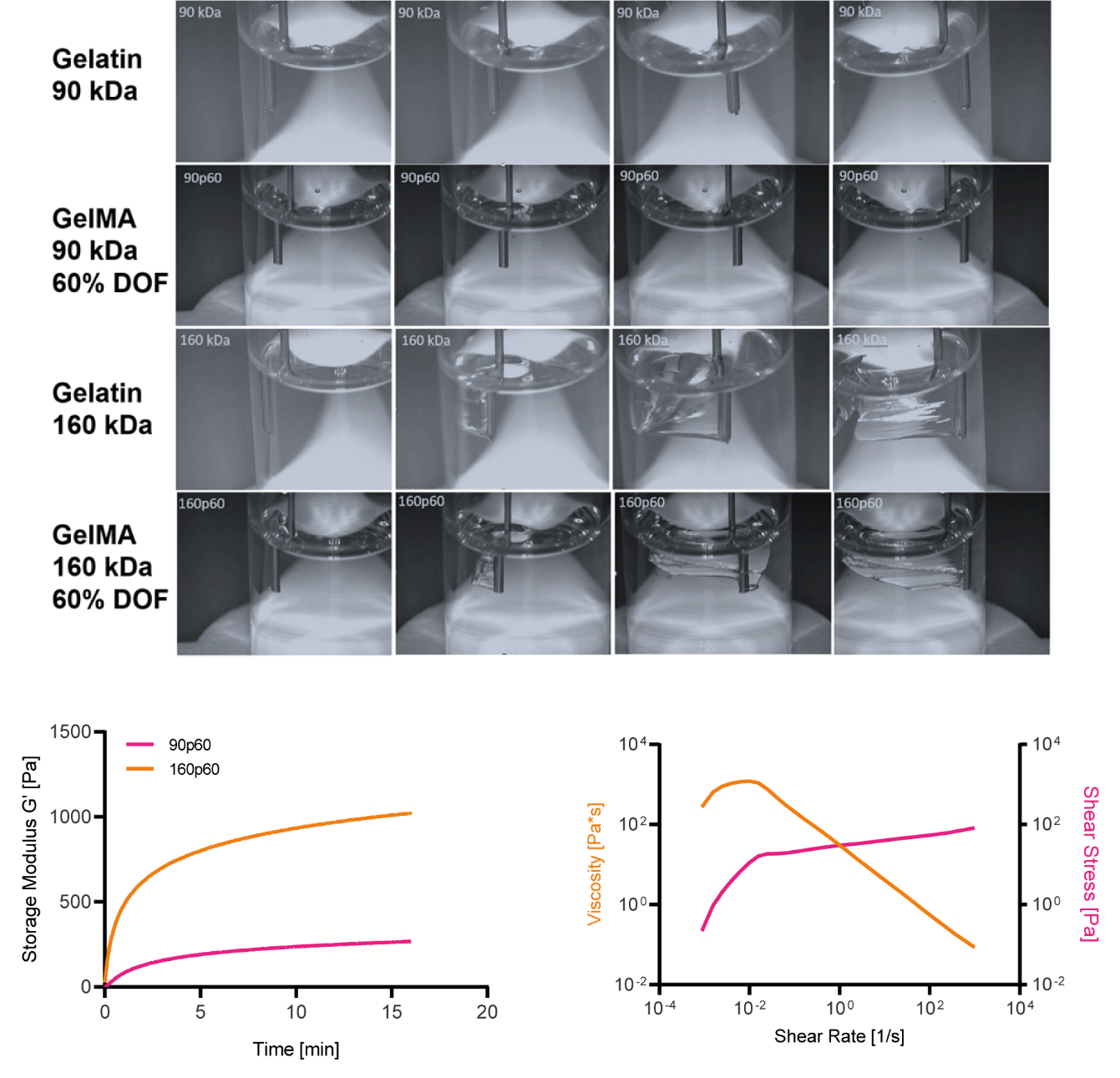


**Supplementary Figure S12:** Self-healing like behaviour for both gelatin and gelMA formulations (5% w/v, room temperature).

90kDa gelatin and GelMAs showed self-healing like properties which avoids scratches creation by the needle translations. The opposite behaviour was observed in 160kDa gelatin, where grooves were indeed created. As the same trend was observed both in gelatin and GelMA formulation, it was proved how the modification didn’t affect the possibility to use the low molecular weight formulation as support bath for embedded extrusion printing.


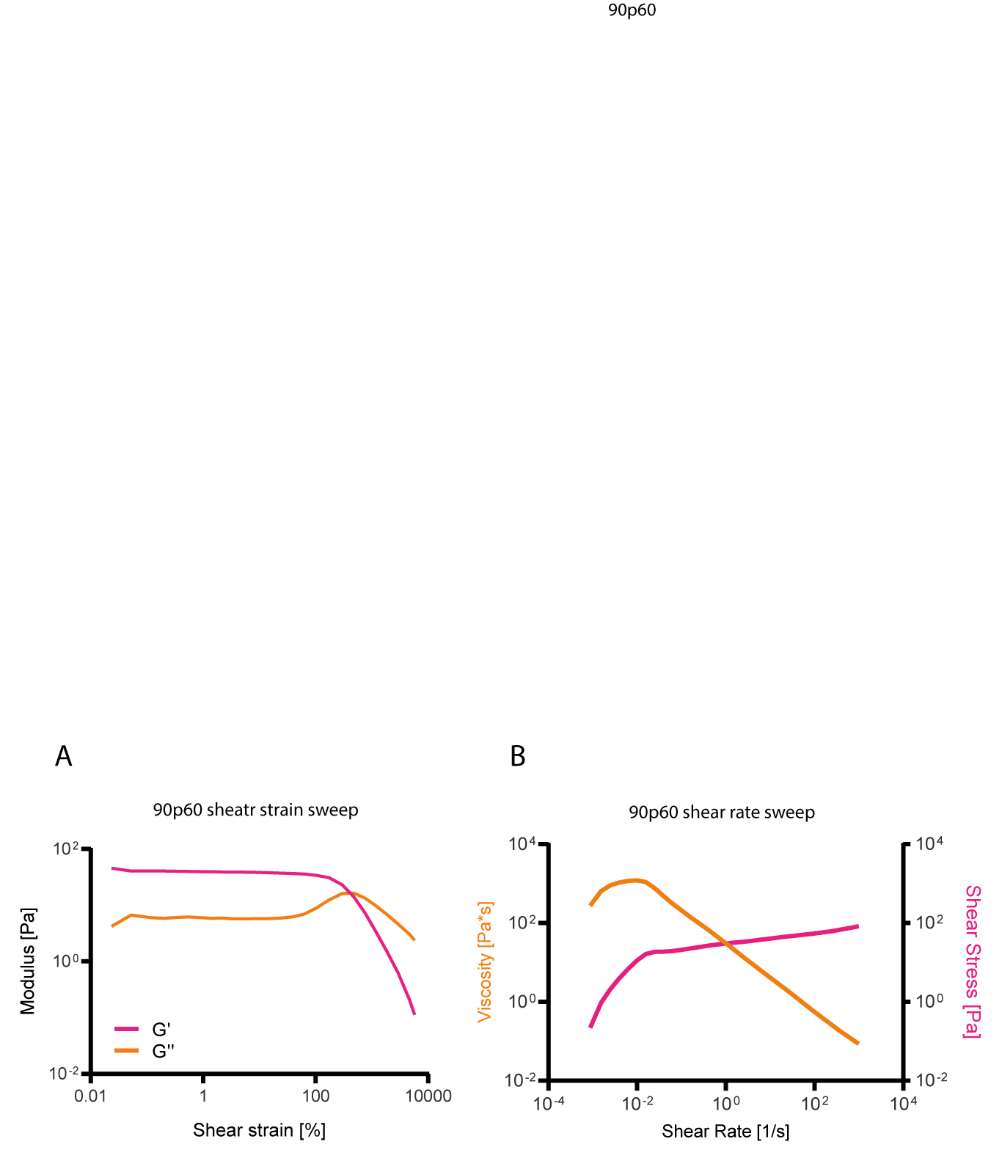


**Supplementary Figure S13:** 90p60 GelMA (5 % w/v, 1x PBS, 0.1 mg mL^-1^ LAP) rheological properties make it suitable as suspension bath for embedded extrusion bioprinting. A) 90p60 shear-yielding with increase in strain (0.037–1000%, 1 Hz) (n=3). B) Shear thinning behaviour observed as the viscosity decreased as the shear strain increased and, under the same conditions, the shear stress increased in a nonlinear fashion.

**8. Multi-wavelength volumetric bioprinter**


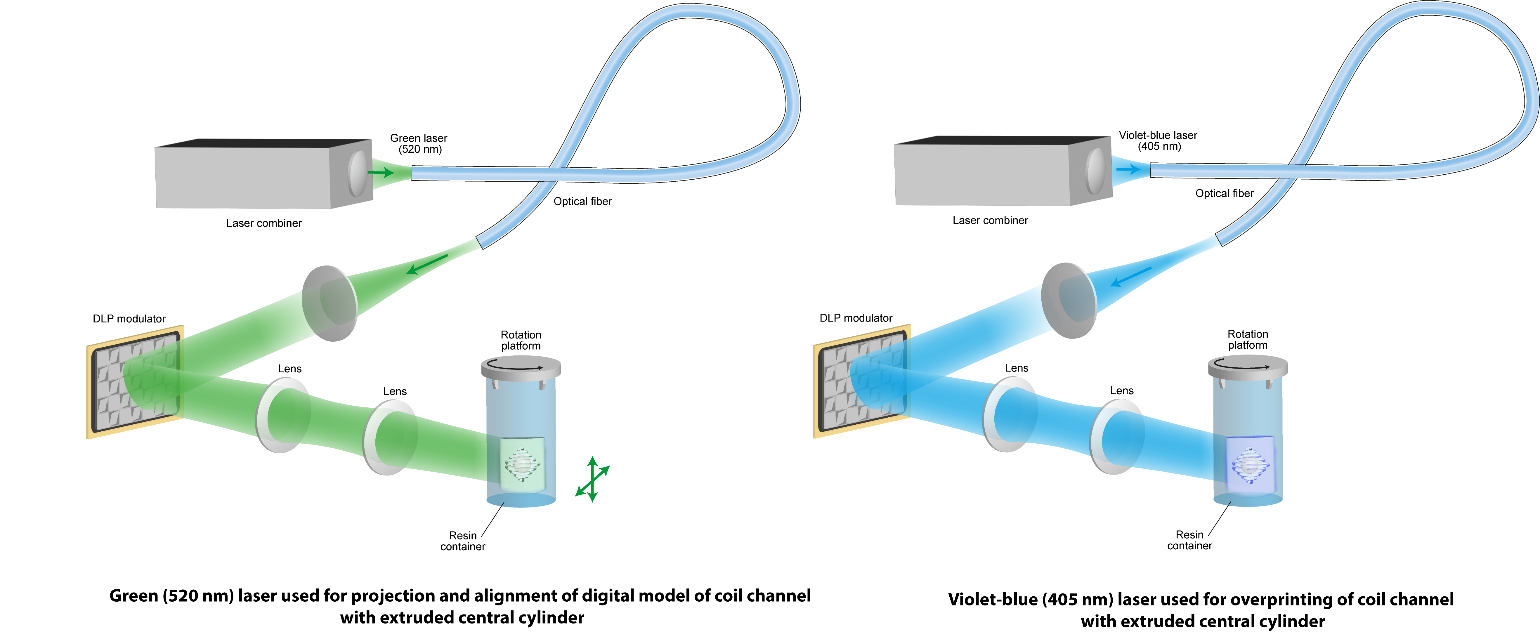


**Supplementary Figure S14:** Multi-wavelength approach for calibration and printing during EmVP.

Green light, far from LAP excitation spectrum, is used for the manual alignment of the vial in the Z axis and XY plane, in order to match the position of the extruded features with the initial angle at which the vial will start to rotate and send, in synchrony, the projections of the object to be overprinted. Subsequently, the volumetric printing process starts using a violet-blue laser line.

**9. Multicellular Embedded Extrusion Volumetric Prtinting**


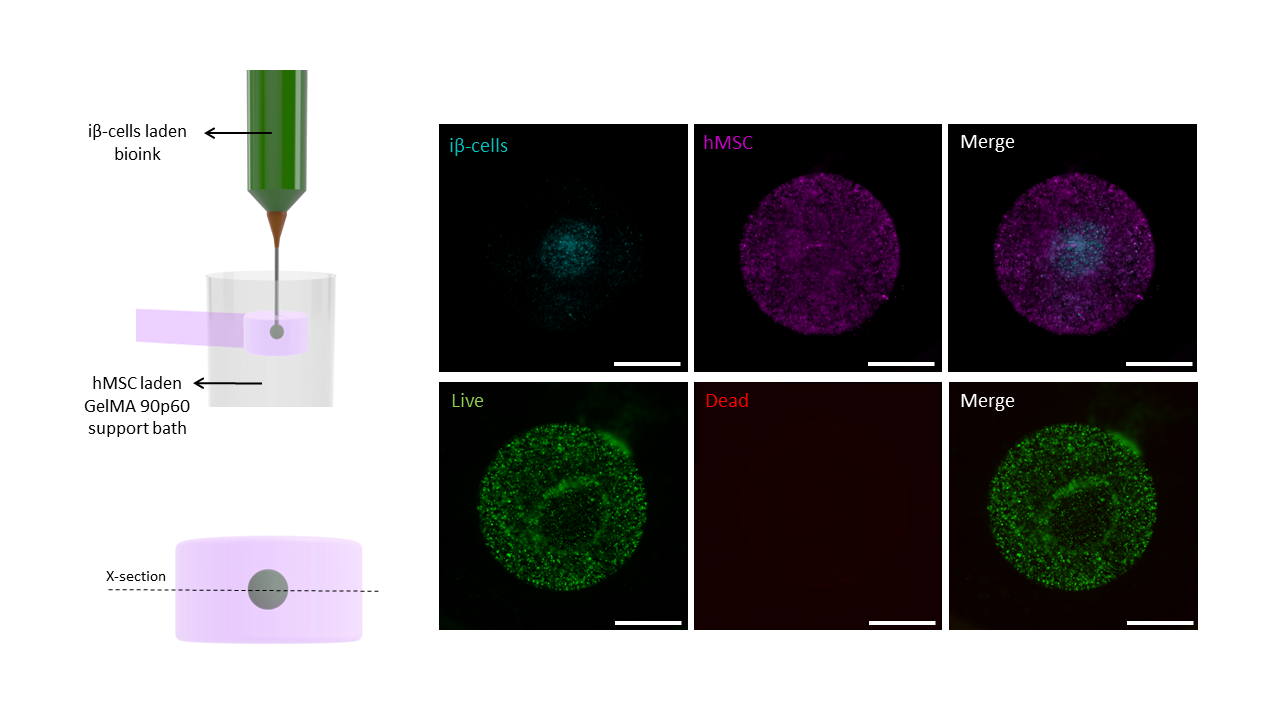


**Supplementary Figure S15: Multicellular Embedded Extrusion Volumetric Printing (EmVP) in 90p60 GelMA as photocrosslinkable support bath.** iβ-cells (10x10^6^/mL) were embedded in a methylcellulose-based bioink and extruded in a hMSC laden (5x10^6^/mL) GelMA 90p60 support bath and volumetrically sculpted to create a multicellular construct. A LIVE/DEAD viability assay was performed after the printing process, showing high viability for both the cell populations, proving the safety of the EmVP process. Scale bar: 2 mm
